# Unveiling local dependencies in accuracy and speed: A mixture hierarchical modeling approach

**DOI:** 10.3758/s13428-026-03125-7

**Published:** 2026-07-24

**Authors:** Hung-Yu Huang

**Affiliations:** Institute of Education, National Cheng Kung University, No.1, University Road, Tainan City, 701 Taiwan

**Keywords:** Hierarchical model, Local dependencies, Mixture models, Item response theory

## Abstract

Hierarchical models (HMs) are commonly used to jointly model response accuracies and response times (RTs). However, their relationship cannot be fully captured by the population-level correlation between ability and speed, as local dependencies may arise and threaten valid inferences about individuals and items. In this study, a mixture-based hierarchical model (Mix-HM) that allows respondents to switch between different pacing speeds and identifies positive and negative item-level dependencies is proposed. Two simulation studies were conducted: Simulation 1 examined parameter recovery effects, and Simulation 2 evaluated the effectiveness of Bayesian information-based criteria in model selection processes. The results showed that the Mix-HM achieved satisfactory parameter recovery effects, whereas the conventional HM yielded biased estimates when local dependencies were present. In addition, the model fit criteria were generally able to correctly identify the true model across most conditions. An empirical analysis conducted using large-scale assessment data further showed that the new model provided an improved degree of fit and more stable parameter estimates, while the conventional HM distorted the relationship between ability and speed. These findings highlight the importance of accounting for the heterogeneity of latent speed and local dependencies when incorporating RTs as collateral information.

## Introduction

With the increasing popularity of computerized testing in educational and cognitive assessments, researchers can now routinely collect not only the correctness of respondents’ answers but also their response times (RTs). Recorded RT data are a valuable source of information, as they help researchers understand the underlying response processes that respondents use during testing and make better inferences about respondents’ underlying abilities (Kyllonen & Zu, 2016; Lee & Chen, 2011; Luce, 1986; van der Linden, 2009; van der Linden et al., 2010). In traditional latent trait measurement models, such as the item response theory (IRT) model (Lord, 1980), respondents’ performance levels are inferred primarily from their response accuracy, and possible differences in response processes (e.g., solution strategies or working speed) are often ignored if no additional information, such as RT, is available.

For decades, efforts in the psychometric field have been devoted to incorporating RT into measurement models. Various psychometric models have been developed to jointly model response accuracy and RT, providing a more fine-grained analysis than traditional latent trait models do (Molenaar et al., 2015a, 2015b; Rouder et al., 2015; van der Linden, 2007; van der Maas et al., 2011). When parallel data are available, where response accuracy and RT per item administered to respondents are collected simultaneously, two separate measurement models should be characterized for response accuracies and RTs. A specific function can then be utilized to account for the dependence between response accuracy and RT for an item. Typically, respondents’ responses to test items can be assumed to follow a specific item response model to construct the correct probability as a function of the intended-measured target and item characteristics. For example, a two-parameter logistic model (Birnbaum, 1968) can be used to fit respondents’ responses to measure latent continuous traits. If latent binary variables are of interest, a diagnostic classification model can be alternatively applied (e.g., the deterministic input–noisy output “AND” gate [DINA] model; Junker & Sijtsma, 2001).

Because raw RTs are skewed with a zero bound, different types of probability density functions have been assumed to describe their characteristic distributions in the literature (e.g., Kang, 2017; Klein Entink et al., 2009b; Loeys et al., 2011; Wang et al., 2013). For ease of interpretation and to make the assumption of linearity and homoscedasticity more plausible, a normal distribution has been used for the converted RT data by log-transforming the raw RTs to align with the response accuracy measurement model within a joint modeling framework (Ferrando & Lorenzo-Seva, 2007; Thissen, 1983; van der Linden, 2006, 2007).

A hierarchical model (HM; Molenaar et al., 2015b; van der Linden, 2007, 2009), which combines an IRT model for response accuracy with a lognormal model for RTs by linking latent ability and speed at the population level, is used for joint analysis in this study. Owing to its theoretical appeal and practical convenience, the HM has been widely applied to both cognitive and noncognitive assessments (e.g., Huang, 2020a; Klein Entink et al., 2009a; Prindle et al., 2016; Wang et al., 2018). By jointly modeling accuracy and RT, the HM enables improved calibration of the target ability and provides a more comprehensive representation of respondents’ response processes during testing (De Boeck & Jeon, 2019; van der Linden et al., 2010). A key assumption underlying this framework is its conditional independence, whereby any observed association between accuracy and RT is assumed to be fully explained by the correlation between the latent ability and speed variables.

However, accumulating evidence suggests that this assumption is often violated in practice, giving rise to local dependency between accuracy and RT within the HM framework (Bolsinova & Maris, 2016; Bolsinova & Molenaar, 2018; Bolsinova & Tijmstra, 2016; Bolsinova et al., 2017a, 2017b; Meng et al., 2015; Molenaar & De Boeck, 2018; Partchev & De Boeck, 2012; Ranger & Ortner, 2012; van der Linden et al., 2010). Following the work of Ranger and Ortner (2012), local dependency can be defined as the residual covariance between the latent continuous response accuracy variable and log-RT after controlling for the higher-level correlation between ability and speed. In other words, even after accounting for the shared variance explained by latent traits, systematic associations may remain in the residuals of accuracy and RT. Such residual dependence cannot be regarded as random noise but instead reflects structured, unmodeled variation that should be explicitly incorporated into the measurement model.

Importantly, local dependency may arise not only from model misspecification but also from systematic behavioral and item-related mechanisms. First, it may reflect respondents’ strategic regulation of response time (Bolsinova & Tijmstra, 2018). Although the conventional speed–accuracy trade-off assumes that faster responses are typically associated with lower accuracy, test-taking behavior often involves dynamic time allocation across items. Respondents may dynamically adjust their speed–accuracy balance throughout the test, such that faster responses may be associated with either higher accuracy (negative dependency) or lower accuracy (positive dependency), with slower responses showing the corresponding opposite pattern, depending on the strategy employed (De Boeck & Jeon, 2019).

Second, item characteristics may induce within-person variation in response speed and thereby contribute to local dependency (Molenaar & De Boeck, 2018). For items requiring lower-level cognitive processing strategies (e.g., memory-based tasks), respondents are more likely to rely on automated knowledge retrieval, leading to faster and more accurate responses. In contrast, items involving higher cognitive demands (e.g., complex problem-solving tasks) typically require greater engagement and controlled processing, resulting in slower but more accurate responses (Bolsinova et al., 2017c; DiTrapani et al., 2016; Goldhammer et al., 2014; Jeon & De Boeck, 2019).

Third, local dependency may reflect idiosyncratic differences among respondents (De Boeck & Jeon, 2019). When a positive dependency is observed, it may arise from cautiousness differences: more careful respondents tend to spend more time to achieve higher accuracy, whereas less engaged respondents respond more quickly with lower accuracy. This pattern is often observed in low-stakes settings. Conversely, when a negative dependency is present, it may reflect cognitive capacity or efficiency differences. Respondents with higher cognitive efficiency levels may produce fast and accurate responses, whereas those with lower efficiency levels exhibit slower and less accurate performance. This pattern is more likely to emerge in high-stakes assessments (for a comprehensive review, see the work of Bolsinova et al., 2017c).

The detection of local dependency between RT and accuracy implies a violation of measurement invariance between the faster and slower classes, potentially compromising the validity of inferences about individual abilities on the basis of performance outcomes. Therefore, models that consider between- and within-person differences in response processes should be developed and utilized to improve the quality of individual measures in tests (Bolsinova et al., 2017c; De Boeck & Jeon, 2019). There are at least two approaches for addressing the possible local dependencies between item response and RT data when the residuals of time and accuracy are found to be correlated in a joint measurement model. Both approaches are based on how a systematic drift in item characteristics occurs concerning RTs and assume that heterogeneity in item responses may arise from speed‒accuracy balance choices and different response processes (Bolsinova et al., 2017c; Molenaar & De Boeck, 2018).

The first approach treats the distinct response processes (i.e., faster and slower responses) as a continuum and utilizes a latent variable model with residual dependencies by introducing local dependency parameters either in the item response function or in the RT function (Bolsinova et al., 2017a; Bolsinova & Tijmstra, 2018; De Boeck et al., 2017; van der Linden et al., 2010). On the other hand, when speed processes are dichotomized into two speed classes (i.e., faster and slower responses) on the basis of diverse response accuracies and time patterns, a mixture modeling approach can alternatively serve as a useful method to investigate the within-person heterogeneity of item characteristics concerning RTs and provide explicit evidence of different response processes from a substantive perspective (De Boeck & Jeon, 2019; Kuijpers et al., 2021; Molenaar & De Boeck, 2018; Molenaar et al., 2016).

Mixture models have been suggested as potentially efficient means of detecting different response processes. These models can simultaneously provide qualitatively different information among latent classes and quantify performance levels among individuals concerning the underlying variable(s) (e.g., Cho & Cohen, 2010; Rost, 1990). Given these advantages, we chose the latent class approach. We developed a new type of mixture model to account for the local dependency between accuracy and RT. This development was carried out within the framework of the traditional HM.

This paper is organized as follows. First, existing mixture models for accuracy and RT are briefly introduced. The newly developed mixture-based HM for local dependency is then elaborated, followed by a comparison with continuous residual-based extensions of the HM. Two simulation studies are subsequently conducted: Simulation 1 evaluates the parameter recovery performance for the proposed model, and Simulation 2 examines the effectiveness of the Bayesian information-based criteria involved in the model selection process. An empirical dataset that includes item responses and RTs is chosen for application demonstration, and the implications of the new model in this example are outlined. Finally, we conclude by discussing the new model’s findings and offering several suggestions and potential model extensions for future research.

## Existing mixture IRT models for responses and RTs

If individuals vary their speed or employ different response strategies during a test, the assumption of conditional independence will be violated in a joint latent trait model. The differences in response patterns across test items can be reflected by the varying patterns in RTs. A mixture model can be used to capture the heterogeneity in item responses and RTs by assuming two latent classes: faster and slower responses (De Boeck & Jeon, 2019). Within the mixture modeling framework, individuals’ performance or response patterns on test items can be characterized as either homogeneous or heterogeneous. When an individual is assumed to exhibit a consistent response process or solution strategy throughout the test (i.e., within-person homogeneous), for example, consistently using a controlled process or problem-solving strategy to respond slowly to all test items or applying quick knowledge retrieval or rapid guessing processes, a *person class* model can be applied. On the other hand, in a *response class* model, within-subject heterogeneity is assumed, meaning that an individual may use one process or strategy for some items and a different one for others.

Meyer (2010) proposed a mixture model for response accuracy and RT that can be treated as a type of person class model, where test-takers can be classified into a regular problem-solving class and a rapid guessing class with respect to their response accuracy and RT patterns, and each class is allowed to have separate item and population parameters in both the Rasch and RT models. As an alternative approach to person class models, Jeon and De Boeck (2019) did not assume two separate models with respect to RTs; rather, they added the RTs as covariates to predict the probability of the two latent classes (i.e., the regular class and knowledge retrieval-based class), which represent the different strategies test-takers apply for solving items. Although the two aforementioned models are capable of detecting different response strategies and different processing speeds to control for the local dependency between RT and accuracy, the assumption of within-person homogeneity with person-level classification appears to be unrealistic because test-takers may switch from one response process (e.g., knowledge retrieval) to another process (e.g., problem solving) depending on the item characteristics and are more likely to seek a balance between speed and accuracy than simply maintaining a constant speed throughout the test (Bolsinova et al., 2017c; De Boeck & Jeon, 2019).

With respect to the response class modeling approach, Wang and Xu (2015) developed a mixture-based hierarchical model that allows within-person heterogeneity to occur in both the item response function and the RT distribution across different items and is capable of differentiating between the two classes, representing the regular solution process and rapid guessing process (also see Wang et al., 2018). A similar approach can be used for the detection of test-takers’ engagement on test items, and the difference in the response processes can be attributed to normal and aberrant behaviors. For the normal response class, the responses and RTs of test-takers attempting an item with solution behavior are assumed to follow a specific IRT model and log-response time distribution function, whereas for the aberrant response class, those attempting an item in a disengaged manner are assumed to rapidly guess the answer or leave the response blank, and the time spent on item responses is not affected by personal or item characteristics (Lu et al., 2020; Ulitzsch et al., 2020). However, test-takers may not always produce responses by rapidly guessing if they decide not to attempt the items; rather, this may be the case where a disengaged test-taker tends to employ partial knowledge or answer the item with partial effort (e.g., Huang, 2020b; Jin & Wang, 2014; San Martín et al., 2006).

Molenaar and colleagues (2016) posited that test-takers’ states for each item can be classified into either faster or slower latent classes by using item-specific latent class variables, but they assumed that the latent class variables are dependent and that test-takers may transit between the two latent classes across items following a first-order Markov transition (also refer to Kuijpers et al., 2021). By focusing on whether systematic within-person speed variations occur across different items, Molenaar et al.’s model uses the same item response function for response accuracy (i.e., the two-parameter logistic model) and the same RT measurement model (i.e., a log-response time normal distribution) but with different sets of item or person population parameters for the two latent classes of faster and slower responses. Although the Markov-based transition mixture model is capable of differentiating latent speed classes for each item and test-taker, the assumption that the state of a test-taker with respect to a certain item is dominantly determined by the state of the previous item may be too restricted to meet real testing situations, and other Markov structures (e.g., higher-order Markov transition) may have effects on the dependency between the states (Marcoulides et al., 2008). Specifically, the associations between adjacent and nonadjacent speed states should be considered in the mixture model, and the dependencies between different states may be captured by random-effect variable(s), as with the case in IRT models (De Boeck & Wilson, 2004). In the next section, we introduce a probabilistic model for speed states and incorporate a random effect to control for individuals’ speed tendencies across items.

Instead of specifying two separate classes for both responses and RTs, Molenaar and De Boeck (2018) developed a response mixture model and applied an identical measurement model to RT data for all respondents. They focused on the detection of faster and slower response classes, and the class membership of one of the two latent classes was regressed on the standardized difference between the observed and expected log-transformed RT. The basic idea behind the proposed mixture model is that whether a person is considered relatively fast or slow depends not only on the time he or she spends on an item but also on the residual time. The greater the observed RT relative to the expected time, the more likely the person’s response belongs to the slower response class, and vice versa. Because the RTs serve as covariates for predicting class membership and are assumed to follow a common probabilistic distributional function, the item-specific latent class variables affect only the responses and not the RTs (Molenaar & De Boeck, 2018).

Although the above response mixture model has the advantages of utilizing response classification and being able to capture the heterogeneity of item characteristics with respect to RTs, some limitations deserve to be noted and discussed. First, it is common for the observed RT to stochastically fluctuate from the expected RT given a log-response time model, and the random fluctuations exhibited by the observed time may compromise the membership classification results. Second, mixtures affect the response accuracy but not the RT; therefore, information about within-person heterogeneity with respect to the response time-relative parameters (i.e., time intensity) of the log-response time model is not accessible. The differences in time intensity between the faster and slower classes may elucidate the investigation of item properties involving, for example, different cognitive processes (Goldhammer et al., 2014). Finally, a common set of mixing parameters is assumed and used for all items in the response mixture model to reduce the computational burden, and the modeling approach may limit the feasibility and utility of the given model (Molenaar & De Boeck, 2018). Because items have different characteristics and require different amounts of time to solve, specifying item- or person-specific mixing parameters to represent the extent to which a given item is related to different latent classes is suggested (Nagy & Ulitzsch, 2022; Wang et al., 2018).

Following the virtues of response classification and focusing on modeling the conditional dependency derived from within-person variability in speed across items, this study aims to develop a new mixture joint model for response accuracy and RT that considers the possible positive or negative local dependencies between accuracy and RT within each item and has the capability to capture the heterogeneity in both item responses and RTs between latent classes, which, to our knowledge, has seldom been explored.

## Model specification

The HM for responses and RTs proposed by van der Linden (2007) integrates an IRT measurement model for accuracy with a linear factor RT model for log-transformed RTs by jointly correlating the latent ability and speed parameters at the top level of the hierarchy. When local dependency between accuracy and RT is suspected in the HM, test-takers with the same latent speed parameter may exhibit different speeds when solving an item. Additionally, the characteristics of a given item may drift across test-takers due to idiosyncratic differences (Bolsinova et al., 2017c). Specifically, a fluctuation in time intensity is observed, where an item may require relatively more or less time for one test-taker than for the average test-taker. In the following, we assume that local dependencies can be explained primarily by two states of response speed, faster- and slower-response favoring classes, as indicated in previous studies (Bolsinova & Molenaar, 2018; Bolsinova et al., 2017a, 2017b; Kuijpers et al., 2021; Molenaar & De Boeck, 2018).

Let ${t}_{ij}$ represent the log-transformed RT of person i on item j, and let ${C}_{ij}$ denote the latent class variable indicating whether test-taker i answered item j, favoring a faster response (${C}_{ij}=2$) or a slower response (${C}_{ij}=1$). We then assume that ${t}_{ij}$ follows a normal distribution with a mean and variance, as given by

1 $$\mathrm{f}\left({t}_{ij}|{\tau}_{i},{C}_{ij}\right)=\mathrm{N}\left({\uplambda}_{jg}-{\uptau}_{i}, {\upsigma}_{res,j}^{2}\right),$$

where ${\uptau}_{i}$ is the latent speed parameter for person *i*, ${\uplambda}_{gj}$ is the time intensity parameter of item *j* for latent class* g* (i.e., ${C}_{ij}=g$ and $g=1 \,\mathrm{a}\mathrm{n}\mathrm{d} \,2$ for the slower- and faster-response favoring classes, respectively), and ${\upsigma}_{res,j}^{2}$ is the residual variance, which is assumed to be homogeneous for the two latent classes. In addition, we constrain ${\uplambda}_{1j}>{\uplambda}_{2j}$ to ensure that the faster-response favoring class has a lower distributional mean than the slower-response favoring class does for each item.

The two-parameter logistic model (2PLM; Birnbaum, 1968) is applied for response accuracy, and the probability of a correct response to item *j* for test-taker *i* belonging to speed class *g* can be formulated as

2 $$\mathrm{P}\left({X}_{ij}=1|{\uptheta}_{i},{C}_{ij},{\upalpha}_{jg},{\upbeta}_{jg}\right)=\frac{\mathrm{e}\mathrm{x}\mathrm{p}\left[{\upalpha}_{jg}\left({\uptheta}_{i}-{\upbeta}_{jg}\right)\right]}{1+\mathrm{e}\mathrm{x}\mathrm{p}\left[{\upalpha}_{jg}\left({\uptheta}_{i}-{\upbeta}_{jg}\right)\right]},$$

where ${\uptheta}_{i}$ is the level of the latent ability of test-taker *i*, and ${\upalpha}_{jg}$ and ${\upbeta}_{jg}$ are the discrimination and difficulty parameters of item* j*, respectively, for latent class *g*. To integrate the 2PLM with the RT model, the latent ability (${\uptheta}_{i}$) and speed (${\uptau}_{i}$) parameters can be assumed to follow a bivariate normal distribution with a zero mean vector and variance‒covariance matrix ${\boldsymbol{\Sigma}}$, where the variance of the latent ability is constrained to 1 for model identification, and other elements in the matrix are freely estimated.

When conditional dependencies arise within the HM, both positive and negative local dependencies should be considered and separated. For this purpose, we create a latent binary indicator to specify whether an item has the effect of positive (i.e., ${q}_{j}=1$) or negative (i.e., ${q}_{j}=0$) dependency. Following Eq. 2, the class-specific item discrimination and difficulty parameters can be further formulated as

3 $${\upbeta}_{j2}={\upbeta}_{j1}+{q}_{j}\times {\updelta}_{j}^{\upbeta }-\left(1-{q}_{j}\right)\times {\updelta}_{j}^{\upbeta }={\upbeta}_{j1}+{\updelta}_{j}^{\upbeta }\times \left(2{q}_{j}-1\right)$$

and

4 $${\upalpha}_{j2}={\upalpha}_{j1}\times {(\frac{1}{{\updelta}_{j}^{\upalpha }})}^{{q}_{j}}\times {{(\updelta }_{j}^{\upalpha })}^{1-{q}_{j}}={\upalpha}_{j1}\times {{(\updelta }_{j}^{\upalpha })}^{1-{2q}_{j}},$$

where ${\upbeta}_{j1}$ and ${\upalpha}_{j1}$ are the item difficulty and discrimination parameters of item* j* for the slower-response favoring class (i.e., ${C}_{ij}=1$), ${\updelta}_{j}^{\upbeta }$ is the difference magnitude parameter in item difficulty between the two classes for item* j* and is constrained to be a positive value, and ${\updelta}_{j}^{\upalpha }$ is the difference magnitude parameter in item discrimination between the two classes for item* j* and is constrained to be positive and larger than 1. Consequently, when positive dependency occurs on item* j* (i.e.,${q}_{j}=1$), the item difficulty parameter of the fast-favored class will be larger than that of the slower-response favoring class (i.e., ${\upbeta}_{j2}>{\upbeta}_{j1}$), and the item discrimination parameter of the faster-response favoring class will be smaller than that of the slower-response favoring class (i.e., ${\upalpha}_{j2}<{\upalpha}_{j1}$), implying that the faster-response favoring class has a lower probability of correct answers and calibrates inferior item discriminating power than the slower-response favoring class does.

On the other hand, if a negative dependency for item* j* is observed (i.e.,${q}_{j}=0$), it implies that the fast-favored class produces more correct answers (i.e., ${\upbeta}_{j2}<{\upbeta}_{j1}$) in response to the given item and can be better discriminated by the item (i.e., ${\upalpha}_{j2}>{\upalpha}_{j1}$) than the slower-response favoring class. Note that whether an item is classified as positive or negative dependence is determined by the *q* latent variable, and the dependency patterns of items are characterized and uncovered entirely via an exploratory approach. Therefore, the underlying local dependency structure can be easily inferred from the collected responses and their corresponding RTs.

There are several justifications for incorporating both positive and negative dependency conditions in the model. First, previous research has shown that cognitive capacity can lead to faster responses with a higher probability of a correct answer, whereas cautiousness is often associated with slower responses that also have a higher likelihood of correctness (Bolsinova et al., 2017c; De Boeck & Jeon, 2019). These varying patterns of dependence are influenced by the characteristics of the test items, which, in turn, shape the examinees’ response strategies.

Second, empirical findings regarding the relationship between RT and ability are mixed. Some studies have found that slower responses contain more diagnostic information about the examinees’ abilities and exhibit higher predictive power in performance assessments (e.g., Coyle, 2003), whereas others have shown that faster responses are often accompanied by a higher probability of correctness and thus are more informative of ability (Bolsinova et al., 2017a). The former supports our modeling assumption for items with positive dependency effects, while the latter aligns with negative dependency effects.

Finally, from a statistical perspective, complex mixture measurement models often encounter challenges such as label switching, which can obscure the interpretation of latent class structures. To address potential label switching in our conditional dependency framework, we adopted parametric constraints to distinguish item dependency patterns, which may help mitigate, this issue, as suggested by Jin et al. (2023). This approach not only improves the identifiability of the model but also allows for a clearer separation between the items exhibiting positive versus negative dependence on the accuracy and RT.

When the local dependencies are considered by introducing the effects of within-person heterogeneity in the item parameters for faster- and slower-response favoring classes, the mixture probability that test-taker *i* correctly answers item *j* can be given by

5 $$\begin{aligned}\mathrm{P}\left({X}_{ij}=1|{\uptheta}_{i},{C}_{ij},{\upalpha}_{jg},{\upbeta}_{jg}\right)&=\sum\nolimits_{{C}_{ij}\in \mathrm{1,2}}\mathrm{P}\left({C}_{ij}=g\right)\times \mathrm{P}\left({X}_{ij}=1|{\uptheta}_{i},{C}_{ij},{\upalpha}_{jg},{\upbeta}_{jg}\right)\\&={\uppi}_{i}\times \mathrm{P}\left({X}_{ij}=1|{\uptheta}_{i},{C}_{ij}=2,{\upalpha}_{j2},{\upbeta}_{j2}\right)+\left({1-\uppi }_{i}\right)\\&\times \mathrm{P}\left({X}_{ij}=1|{\uptheta}_{i},{C}_{ij}=1,{\upalpha}_{j1},{\upbeta}_{j1}\right), \end{aligned}$$

where $\mathrm{P}\left({C}_{ij}=g\right)$ is the mixing probability, which gives the probability that test-taker *i*’s response to item *j* follows a 2PLM specified for the faster-response favoring class (when *g* = 2) via $\mathrm{P}\left({C}_{ij}=2\right)={\uppi}_{i}$ or slower-response favoring class (when* g* = 1) via $\mathrm{P}\left({C}_{i}=1\right)=1-{\uppi}_{i}$, and the other parameters are as previously defined. The person-level ${\uppi}_{i}$ parameter is used to indicate whether a response to an item is governed by faster- or slower-response favoring tendencies and can be assumed to be random across test-takers. A conjugate prior of the beta distribution is a reasonable candidate for the distribution of ${\uppi}_{i}$ (DeCarlo, 2020); however, to be integrated with the framework of generalized linear models with maximum flexibility, the logistic-normal model can be implemented for specifying the distribution of ${\uppi}_{i}$ (Aitchison & Shen, 1980; DeCarlo, 2021a, 2021b), which is given by

6 $$\mathrm{P}\left({C}_{ij}=2\right)={\uppi}_{i}\equiv \frac{\mathrm{e}\mathrm{x}\mathrm{p}\left({\upeta}_{0}+{\upeta}_{1}\times {\upxi}_{i}\right)}{1+\mathrm{e}\mathrm{x}\mathrm{p}\left({\upeta}_{0}+{\upeta}_{1}\times {\upxi}_{i}\right)}.$$

Here, ${\upeta}_{0}$ and ${\upeta}_{1}$ are the location and scale parameters, respectively, which determine the shape of the ${\uppi}_{i}$ distribution (for more details, see DeCarlo, 2021a, p. 31). The ${\upxi}_{i}$ parameter represents person-specific adaptability, capturing the within-person heterogeneity in the tendency for faster-favoring responses, influenced by the individual’s strategy in balancing speed and accuracy (Bolsinova et al., 2017c; De Boeck & Jeon, 2019).

For model identification, the ${\upxi}_{i}$ parameter is assumed to follow a standard normal distribution. A higher ${\upxi}_{i}$ value indicates a greater likelihood that a test-taker will respond to any given item more quickly. Note that a more flexible specification could allow the response mixing probability to vary across both different persons and items (e.g., ${\uppi}_{ij}$​), thereby capturing the additional heterogeneity that is driven by item characteristics. However, such an extension would substantially increase the complexity of the model and is beyond the scope of the current study.

It can be further assumed that the probability of a correct answer for a specific item is related to the probability of being in the faster- or slower-response favoring class in the mixture hierarchical model by constructing the latent regression of ${\upxi}_{i}$ on ${\uptheta}_{i}$, which can be formulated as follows:

7 $${\upxi}_{i}={\upomega \uptheta }_{i}+{\upvarepsilon}_{i},$$

where ω is the regression coefficient used to associate the two latent trait variables and where ${\upvarepsilon}_{i}$ is the prediction residual for person *i* and ${\upvarepsilon}_{i}\sim \mathrm{N}\left(0, 1-{\upomega }^{2}\right)$. Notably, the ω parameter indicates the association of latent proficiency with the adaptability that test-takers possess and captures the intraindividual speeding tendency for each item. In contrast, the conventional HM considers the overall effective speed of a test-taker, with the higher-level structure reflecting an overall relationship between ability and speed across all items (Bolsinova & Tijmstra, 2018; Mutak et al., 2024).

By combining the 2PLM for accuracy responses and the lognormal model for RTs within a mixture framework, the marginal likelihood of the observed data can be expressed as follows:

8 $$\begin{aligned}\mathcal{L}\left({\mathbf{X}},{\mathbf{t}};\,{\mathbf{v}}\right)={\prod}_{i=1}^{N}{\sum}_{{C}_{ij}\in \mathrm{1,2}}P\left({C}_{ij}=g\right)\times \iint {\prod}_{j=1}^{J}P{\left({X}_{ij}=1\left|{\uptheta}_{i},{C}_{ij},{\upalpha}_{jg},{\upbeta}_{jg}\right.\right)}^{{x}_{ij}}\\\times P{\left({X}_{ij}=0\left|{\uptheta}_{i},{C}_{ij},{\upalpha}_{jg},{\upbeta}_{jg}\right.\right)}^{1-{x}_{ij}}\times f\left({t}_{ij}\left|{\uptau}_{i}\right.,{C}_{ij},{\lambda}_{jg}\right)g\left({\uptheta}_{i},{\uptau}_{i}\right)d\uptheta\, d\uptau , \end{aligned}$$

where ${\boldsymbol{\upnu}}$ denotes the full vector of model parameters, $\mathrm{g}\left({\uptheta}_{i},{\uptau}_{i}\right)$ is the bivariate normal density function that describes the person-level ability and speed parameters, and the remaining components are as previously defined.

## Comparison with continuous residual-based extensions of the hierarchical model

A natural question for researchers and practitioners is how the proposed mixture-based hierarchical model (Mix-HM) differs from alternative approaches that account for conditional dependency between response accuracy and RT. In particular, it is informative to examine how the results and substantive interpretations obtained from Mix-HM compare with those derived from models that treat within-person variation in response speed as a continuous process rather than a class-based phenomenon.

Among the various continuous latent variable models that accommodate residual dependencies between accuracy and RT, we focus on the continuous-variable extension of the hierarchical model proposed by Bolsinova et al. (2017a), which is hereafter referred to as the CV-HM. This model is selected for comparison purposes because it shares an important conceptual similarity with the proposed Mix-HM: both approaches allow item characteristics to systematically vary as a function of within-person response speed heterogeneity. The key difference between these models lies in how such heterogeneity is conceptualized. The CV-HM models the effect of speed through a continuous standardized log-RT residual, whereas the Mix-HM assumes that responses may arise from distinct latent speed classes representing faster- and slower-response processes. By contrasting these two perspectives—continuous and class-based viewpoints—we aim to clarify their conceptual distinctions and demonstrate how a class-based representation may provide complementary insights into within-person speed heterogeneity.

In the CV-HM, the item response function for accuracy is specified as a function of the standardized log-transformed RT residuals. Consequently, the greater the deviation of an observed log-RT is from the model-expected value, the larger the predicted shifts in the item difficulty and discrimination parameters for that particular person–item interaction. This formulation implies that the estimated item parameters are highly sensitive to the magnitudes of RT deviations and may be substantially influenced by extreme residual values. Because RTs often contain stochastic fluctuations, the resulting item parameter adjustments may become unstable or difficult to interpret when large residual deviations occur.

Moreover, the prediction functions linking the RT residuals to the item difficulty and discrimination parameters are specified without explicit prediction residual terms. In other words, the predicted item parameter shifts are assumed to be deterministic functions of the standardized RT residuals. This structure resembles the limitation noted in models such as the linear logistic test model (LLTM), where item parameters are fully determined by the predictors without accounting for unexplained variations (De Boeck & Wilson, 2004). As a result, random fluctuations in the RTs may be absorbed into other variance components of the model.

In contrast, the proposed Mix-HM adopts a simpler and more parsimonious representation of within-person speed heterogeneity. Rather than allowing item parameters to vary continuously for every person–item combination as in the CV-HM, the model assumes that responses may arise from two latent speed states representing slower- and faster-response processes. Under this framework, item parameters are defined at the class level, which facilitates a clearer interpretation of local positive or negative dependencies between accuracy and RT while reducing the sensitivity of the parameter estimates to extreme RT residuals. Importantly, this mixture representation should not be interpreted as an implication that response speeds are inherently discrete. Instead, the latent classes provide a parsimonious approximation of the heterogeneous response processes that may arise from the different speed–accuracy strategies adopted by test-takers.

Furthermore, the proposed Mix-HM is not a purely class-based model. As specified in Eq. 6, the mixing probability is modeled through a continuous latent variable ${\upxi}_{i}$​, which quantifies each respondent’s tendency toward faster-favored responses. This parameter represents person-specific adaptability and captures the within-person heterogeneity for striking a balance between speed and accuracy across different items. Consequently, the Mix-HM incorporates both a discrete representation of response states (slow vs. fast) and a continuous latent tendency governing the probability of belonging to these states. This hybrid structure allows the model to approximate heterogeneous response processes while maintaining strong interpretability and parsimony. A similar modeling advantage has been noted in higher-order cognitive diagnostic models (de la Torre & Douglas, 2004), which also combine discrete classifications with a continuous latent trait to obtain flexible yet interpretable representations of individual differences.

As indicated in the literature on conditional dependence (Bolsinova et al., 2017c), dependencies between response accuracies and RTs may arise from different sources. One possibility concerns homogeneous response processes, where individuals rely on a similar underlying strategy but differ in their levels of cautiousness, cognitive capacity, or effort allocation. Another possibility involves heterogeneous response processes, where respondents employ qualitatively different solving strategies during the test. The former situation can often be addressed through both mixture-based and continuous latent variable-based modeling approaches, as both frameworks are capable of capturing systematic variation in the relationship between accuracy and RT. In contrast, the latter situation involves distinguishing between different types of response processes, such as relatively fast responses driven by automatic retrieval versus slower responses that are associated with more controlled processing schemes.

In their systematic review of models for addressing RTs and response processes in cognitive tests, De Boeck and Jeon (2019) noted that mixture-based models and continuous latent-variable models may yield broadly comparable findings when addressing conditional dependence within the hierarchical modeling framework. Nevertheless, they emphasized that mixture-based models offer a practical advantage when the research goal is to identify qualitatively distinct response processes. For example, mixture formulations have been used to differentiate engaged responses from rapid guessing or cheating (Wang et al., 2018), as well as to distinguish regular problem-solving schemes from knowledge-retrieval strategies (Jeon & De Boeck, 2019). Similarly, Molenaar et al. (2016) employed a Markov-transition mixture model to explore how test-takers alternate between slower and faster response states during a cognitive test, and they further demonstrated how such classifications can be linked to theoretically meaningful strategy types, such as those described in Siegler’s (1981) model of multiple problem-solving strategies.

Overall, continuous-variable–based approaches and mixture-based approaches serve different analytical purposes when modeling conditional dependency between response accuracy and RT. Continuous models provide a detailed representation of individual variation in speed–accuracy relationships, whereas mixture formulations are particularly useful when the research interest involves identifying qualitatively different response processes. The proposed Mix-HM integrates elements of both perspectives by combining a latent response-state classification strategy with a continuous adaptability parameter governing the probability of faster- or slower-favored responses. In this sense, the model retains the interpretability of response-process classification while simultaneously capturing continuous individual differences in response tendencies. To further illustrate these distinctions, the empirical study presented later in this article includes a comparative analysis of the Mix-HM and the CV-HM.

## Simulation 1: Parameter recovery assessment of the Mix-HM

### Design

Two simulation studies were conducted. Simulation 1 examined the parameter recovery performance of the Mix-HM and evaluated the consequences of using the conventional HM to fit data generated from the Mix-HM, particularly with respect to person parameter estimation. Simulation 2 investigated the effects of information-based model fit criteria on the ability to correctly identify the data-generating model as the best-fitting model.

In Simulation 1, the proposed Mix-HM was used to generate response accuracy and RT data. Both the true Mix-HM and the conventional HM were fitted to the generated datasets to evaluate their parameter recovery effects and to examine the consequences of ignoring the mixture structure arising from heterogeneous response paces. The simulation conditions varied in terms of the sample size (500 and 1,000 examinees) and test length (20 and 40 items). In addition, the proportions of positive- and negative-dependency items were manipulated. We considered three conditions: equal representation (1:1), a negative-dependency–dominant condition (3:1), and a positive-dependency–dominant condition (3:1). All other conditions were held constant.

Due to the assumed local dependencies between responses and RTs, and influence of two states, i.e., faster- and slower-favored responses, we needed to specify two distinct sets of item parameters for the measurement models of response accuracy and RT for the faster- and slower-favored classes. First, a bivariate normal distribution with a mean vector of (0, 4) and a variance‒covariance matrix of $$\left[\begin{array}{cc}1& {\uprho}_{\beta \lambda }\sqrt{1/3}\\ {\uprho}_{\beta \lambda }\sqrt{1/3}& 1/3\end{array}\right]$$ was used to generate the item difficulty β parameters and item time‒intensity λ parameters, respectively. The correlation parameter ${\uprho}_{\beta \lambda }$ was set to 0.65, according to previous studies (Huang, 2020a; van der Linden, 2009). The time residual variances were randomly generated within a range of 0.40 to 0.67, as utilized in a study by Wang et al. (2018).

To generate the faster- and slower-favored latent classes in the lognormal RT model, the item time–intensity parameters for the slower-favored class were directly set as the generated λ values (i.e., ${\uplambda}_{j1}={\uplambda}_{j}$), whereas those for the faster-favored class were obtained by subtracting one unit from the same values (i.e., ${\uplambda}_{j2}={\uplambda}_{j1}-1$). This configuration produced a systematic difference in speed-related parameters across the two latent classes, similar to the approach used in prior work (DeCarlo, 2021b).

For the accuracy component, the generated item difficulty parameters were applied to the slower-favored class (i.e., ${\upbeta}_{j1}$​), and the item difficulty difference between the faster- and slower-favored classes (i.e., ${\updelta}_{j}^{\upbeta }$​) was set to 0.5 for all items. The latent binary variable ${q}_{j}$​, indicating the direction of dependency, was generated to satisfy the specified composition condition (1:1, 3:1 negative-dominant, and 3:1 positive-dominant). Given ${q}_{j}$​, the item difficulty parameter for the faster-favored class (i.e., ${\upbeta}_{j2}$​) was generated by adding ${\updelta}_{j}^{\upbeta }$​ when a positive dependency was present and subtracting ${\updelta}_{j}^{\upbeta }$​ when a negative dependency was present.

Regarding the item discrimination parameters, the ${\upalpha}_{j1}$ parameter for the slower class was generated from a uniform distribution between 0.5 and 1.5, and the difference in item discrimination between the two latent classes (i.e., ${\updelta}_{j}^{\upalpha }$) was set to 1.5 for all items. Following the generated ${\upalpha}_{j1}$, ${\updelta}_{j}^{\upalpha }$, and ${\mathrm{q}}_{j}$ values, the ${\upalpha}_{j2}$ parameter for the faster-favored class was calculated according to Eq. 4: for item positive dependency, ${\upalpha}_{j1}\times \frac{1}{{\updelta}_{j}^{\upalpha }}$; for item negative dependency, ${\upalpha}_{j1}\times {\updelta}_{j}^{\upalpha }$. The ranges of the generated α and β parameters were consistent with those commonly used in the literature on latent differential item functioning (e.g., Huang, 2017) and were broadly comparable to the settings employed in studies of response behavior differences between faster and slower response classes (Kuijpers et al., 2021; Molenaar & De Boeck, 2018; Molenaar et al., 2016), though the specific values were only approximately aligned.

The person parameters of latent ability and speed were sampled from a bivariate normal distribution with a zero mean vector and a variance‒covariance matrix of $$\left[\begin{array}{cc}1& {\uprho}_{\theta \tau }\sqrt{.25}\\ {\uprho}_{\theta \tau }\sqrt{.25}& .25\end{array}\right]$$, with ${\uprho}_{\theta \tau }$ set to 0.5. This design is consistent with that used in a study conducted by Lu et al. (2020). In addition, we set $\left({\upeta}_{0}, {\upeta}_{1}\right)=(0, 0.6)$ to enforce the distribution of ${\uppi}_{i}$ to appear normal, aiming to make the probability of belonging to one of the two latent classes nearly equal across items for each respondent (see DeCarlo, 2021a). Finally, the regression coefficient ω was set to a negative value of − 0.80 on the basis of findings from real data analysis, assuming that higher-proficiency test-takers are more likely to have a lower probability of belonging to the faster-favored class than their lower-proficiency counterparts are (DeCarlo, 2021b).

### Analysis

All the model parameters were calibrated via the JAGS package with Bayesian methods (Plummer, 2017). Before applying Bayesian estimation, a set of prior distributions for the model parameters must be specified. A normal prior distribution with a mean of zero and a variance of 4 was used for both the item difficulty parameters of the slower class (i.e., ${\upbeta}_{j1}$) and the location parameter (i.e., ${\upeta}_{0}$). A lognormal distribution with a mean of zero and a variance of 1 was used for the item discrimination parameters of the slower class (i.e., ${\upalpha}_{j1}$), the difference magnitude parameter in item difficulty (i.e., ${\updelta}_{j}^{\upbeta }$), and the scale parameter (i.e., ${\upeta}_{1}$). The same lognormal prior was set for the difference magnitude parameter in item discrimination (i.e., ${\updelta}_{j}^{\upalpha }$) but with a truncated distribution between zero and 1. A gamma prior distribution with both hyperparameters equal to 0.01 was specified for both the inverse of the log-transformed response time residual variances (i.e., ${\upsigma}_{j}^{2}$) and the inverse of the variance of the latent speed parameters (i.e., ${\upsigma}_{\tau }^{2}$). A uniform distribution ranging from − 0.99 to 0.99 was set for both the correlation between the latent ability and speed parameters (i.e., ${\uprho}_{\theta \tau }$) and the regression coefficient parameter (i.e., ω). We assumed that the latent binary indicator (i.e., ${q}_{j}$) followed a Bernoulli distribution with a probability of ${\mathrm{P}}_{q}$ and set a beta prior with both hyperparameters equal to 1 for ${\mathrm{P}}_{q}$.

Finally, to avoid the label-switching problem that commonly emerges in Bayesian mixture models (Meyer, 2010), we used a normal prior distribution with a mean of 4 and a variance of 4 for the item time‒intensity parameters and constrained the time‒intensity parameters of the faster-favored class to be smaller than those of the slower-favored class. This approach is similar to that utilized by Molenaar and De Boeck (2018). The priors were applied to both the simulations and the empirical analysis and set in accordance with or similar to those in previous studies and empirical findings (e.g., Cho & Cohen, 2010; Cohen & Bolt, 2005; Huang, 2016, 2017; Wang et al., 2018).

Bayesian estimation for each condition was conducted by using three independent Markov chains with random initial values. Each chain was run for 20,000 iterations, with the first 5,000 iterations discarded as burn-in. To reduce autocorrelation, every fifth iteration was retained (i.e., thinning = 5). In our analyses, convergence was monitored by using the multivariate potential scale reduction factor (MPSRF; Brooks & Gelman, 1998), and convergence was considered satisfactory when the MPSRF across the three chains approached 1.1.

Each simulation condition was replicated 50 times. This number of replications was selected to balance computational feasibility with estimation stability. To ensure that 50 replications was sufficient, a random subset of simulation conditions was replicated beyond 50. The resulting variation in parameter recovery indices was minimal, indicating that 50 replications per condition was adequate for producing stable and reliable results.

For the structural parameters, bias and root mean square error (RMSE) were computed to evaluate the recovery accuracy of each estimator across replications. The accuracy of person parameter estimates was assessed using the root mean square difference (RMSD), defined as the square root of the mean squared difference between the true and estimated latent trait values across individuals within each replication.

To evaluate the recovery of the latent class structure, correct classification rates (CCR) were computed. In the proposed Mix-HM, latent classes are defined at multiple levels, including the response-level latent class variable representing faster- and slower-response processes and the item-level latent indicator capturing positive and negative dependency patterns. Accordingly, CCR was calculated for both the response-level latent class assignments and the item-level dependency indicators for each dataset. The mean CCR across replications served as a summary measure of the model’s ability to accurately recover the underlying latent class structure and dependency patterns, providing a direct and interpretable index of classification performance alongside parameter recovery indices.

### Results

To prevent excessive data presentation and to respect space constraints, the recovery performance of the item parameters was evaluated using mean bias and RMSE across items. Tables 1, 2, 3 present the results obtained for the balanced, negative-dominant, and positive-dominant dependency conditions, respectively. The simulation results indicate that the bias values were consistently close to zero and that the RMSE values were relatively small and within an acceptable range. Increasing the sample size generally reduced the RMSE values, whereas increasing the test length yielded a less consistent pattern. Across the three dependency composition conditions (balanced, negative-dominant, and positive-dominant), the results remained largely comparable, suggesting that varying the proportions of positive- and negative-dependency items had only a minimal impact on the parameter recovery performance.

**Table 1:** Statistical summary of parameter recovery under the balanced dependency condition in Simulation 1

Sample size	500				1,000			
Test length	20		40		20		40	
Criterion	Bias	RMSE	Bias	RMSE	Bias	RMSE	Bias	RMSE
Parameter								
${\upalpha}_{j1}$	0.014	0.214	0.033	0.243	0.030	0.167	0.005	0.157
${\upbeta}_{j1}$	0.022	0.207	0.035	0.239	0.017	0.145	0.001	0.179
${\updelta}_{j}^{\upalpha }$	− 0.063	0.124	− 0.040	0.115	− 0.035	0.124	− 0.012	0.110
${\updelta}_{j}^{\upbeta }$	0.028	0.092	0.029	0.091	0.020	0.114	0.022	0.110
${\uplambda}_{j1}$	− 0.046	0.092	− 0.030	0.080	− 0.022	0.056	− 0.016	0.050
${\uplambda}_{j2}$	0.056	0.099	0.041	0.085	0.029	0.062	0.016	0.047
${\upsigma}_{res,j}^{2}$	− 0.065	0.166	− 0.049	0.150	− 0.034	0.114	− 0.029	0.101
${\upsigma}_{\tau }^{2}$	− 0.012	0.025	− 0.003	0.017	0.001	0.012	− 0.002	0.010
${\uprho}_{\theta \tau }$	− 0.022	0.040	− 0.012	0.028	− 0.007	0.021	− 0.004	0.015
ω	0.065	0.123	− 0.009	0.052	0.005	0.073	− 0.001	0.044
${\upeta}_{0}$	0.009	0.065	0.011	0.059	− 0.002	0.037	− 0.014	0.038
${\upeta}_{1}$	− 0.003	0.095	0.000	0.065	− 0.004	0.062	− 0.005	0.030
Classification	CCR							
${q}_{j}$	0.900		0.910		0.957		0.955	
${C}_{ij}$	0.817		0.828		0.821		0.831	

${\upalpha}_{j1}$ is the item discrimination parameter of the slower-response favoring class; ${\upbeta}_{j1}$ is the item difficulty parameter of the slower-response favoring class; ${\updelta}_{j}^{\upalpha }$ and ${\updelta}_{j}^{\upbeta }$ are the difference magnitude parameters between the two classes for item discrimination and difficulty, respectively; ${\uplambda}_{j1}$ and ${\uplambda}_{j2}$ are the time intensity parameters for the slower- and faster-response favoring classes, respectively; ${\upsigma}_{res,j}^{2}$ is the RT residual variance; ${\upsigma}_{\tau }^{2}$ is the variance of the speed parameters; ${\uprho}_{\theta \tau }$ is the correlation between the ability and speed parameters; ω is the regression weight of the ability parameter on the adaptability parameter; ${\upeta}_{0}$ and ${\upeta}_{1}$ are the location and scale parameters, respectively; ${q}_{j}$ is the latent item indicator variable; and ${C}_{ij}$ is the latent class variable of a person’s response to an item. The positive- and negative-dependency items were equally distributed (1:1)

**Table 2:** Statistical summary of parameter recovery under the negative-dominant dependency condition in Simulation 1

Sample size	500				1,000			
Test length	20		40		20		40	
Criterion	Bias	RMSE	Bias	RMSE	Bias	RMSE	Bias	RMSE
Parameter								
${\upalpha}_{j1}$	− 0.035	0.212	− 0.014	0.207	− 0.023	0.147	− 0.007	0.151
${\upbeta}_{j1}$	0.033	0.202	0.056	0.249	0.023	0.153	0.030	0.189
${\updelta}_{j}^{\upalpha }$	− 0.071	0.136	− 0.041	0.111	− 0.023	0.115	− 0.006	0.109
${\updelta}_{j}^{\upbeta }$	0.022	0.099	0.027	0.090	0.030	0.123	0.018	0.107
${\uplambda}_{j1}$	− 0.056	0.108	− 0.044	0.090	− 0.027	0.065	− 0.024	0.055
${\uplambda}_{j2}$	0.055	0.104	0.049	0.093	0.020	0.060	0.014	0.051
${\upsigma}_{res,j}^{2}$	− 0.071	0.173	− 0.069	0.163	− 0.038	0.116	− 0.028	0.101
${\upsigma}_{\tau }^{2}$	− 0.013	0.022	− 0.006	0.016	− 0.005	0.014	− 0.003	0.013
${\uprho}_{\theta \tau }$	− 0.042	0.074	− 0.028	0.046	− 0.015	0.044	− 0.011	0.032
ω	0.055	0.146	0.017	0.085	0.033	0.059	0.003	0.042
${\upeta}_{0}$	− 0.020	0.072	− 0.011	0.061	0.006	0.055	− 0.010	0.039
${\upeta}_{1}$	− 0.013	0.107	− 0.017	0.070	0.019	0.079	− 0.007	0.037
Classification	CCR							
${q}_{j}$	0.915		0.879		0.953		0.945	
${C}_{ij}$	0.807		0.820		0.813		0.823	

${\upalpha}_{j1}$ is the item discrimination parameter of the slower-response favoring class; ${\upbeta}_{j1}$ is the item difficulty parameter of the slower-response favoring class; ${\updelta}_{j}^{\upalpha }$ and ${\updelta}_{j}^{\upbeta }$ are the difference magnitude parameters between the two classes for item discrimination and difficulty, respectively; ${\uplambda}_{j1}$ and ${\uplambda}_{j2}$ are the time intensity parameters for the slower- and faster-response favoring classes, respectively; ${\upsigma}_{res,j}^{2}$ is the RT residual variance; ${\upsigma}_{\tau }^{2}$ is the variance of the speed parameters; ${\uprho}_{\theta \tau }$ is the correlation between the ability and speed parameters; ω is the regression weight of the ability parameter on the adaptability parameter; ${\upeta}_{0}$ and ${\upeta}_{1}$ are the location and scale parameters, respectively; ${q}_{j}$ is the latent item indicator variable; and ${C}_{ij}$ is the latent class variable of a person’s response to an item. The negative-dependency items were more prevalent than the positive-dependency items (3:1)

**Table 3:** Statistical summary of parameter recovery under the positive-dominant dependency condition in Simulation 1

Sample size	500				1,000			
Test length	20		40		20		40	
Criterion	Bias	RMSE	Bias	RMSE	Bias	RMSE	Bias	RMSE
Parameter								
${\upalpha}_{j1}$	0.020	0.230	0.056	0.227	0.006	0.159	0.027	0.165
${\upbeta}_{j1}$	0.040	0.235	0.019	0.264	0.031	0.171	0.014	0.193
${\updelta}_{j}^{\upalpha }$	− 0.066	0.130	− 0.051	0.119	− 0.037	0.119	− 0.017	0.108
${\updelta}_{j}^{\upbeta }$	0.029	0.090	0.027	0.081	0.032	0.108	0.028	0.103
${\uplambda}_{j1}$	− 0.053	0.104	− 0.044	0.091	− 0.027	0.064	− 0.024	0.055
${\uplambda}_{j2}$	0.053	0.101	0.049	0.093	0.020	0.061	0.014	0.051
${\upsigma}_{res,j}^{2}$	− 0.067	0.169	− 0.069	0.163	− 0.039	0.115	− 0.028	0.100
${\upsigma}_{\tau }^{2}$	− 0.027	0.041	− 0.006	0.016	− 0.005	0.013	− 0.003	0.012
${\uprho}_{\theta \tau }$	− 0.042	0.072	− 0.025	0.048	− 0.015	0.040	− 0.009	0.030
ω	0.063	0.117	0.014	0.087	0.032	0.067	− 0.004	0.061
${\upeta}_{0}$	− 0.017	0.068	− 0.009	0.060	0.006	0.060	− 0.010	0.039
${\upeta}_{1}$	− 0.003	0.105	− 0.009	0.074	0.025	0.083	− 0.004	0.037
Classification	CCR							
${q}_{j}$	0.958		0.910		0.983		0.948	
${C}_{ij}$	0.808		0.820		0.813		0.824	

${\upalpha}_{j1}$ is the item discrimination parameter of the slower-response favoring class; ${\upbeta}_{j1}$ is the item difficulty parameter of the slower-response favoring class; ${\updelta}_{j}^{\upalpha }$ and ${\updelta}_{j}^{\upbeta }$ are the difference magnitude parameters between the two classes for item discrimination and difficulty, respectively; ${\uplambda}_{j1}$ and ${\uplambda}_{j2}$ are the time intensity parameters for the slower- and faster-response favoring classes, respectively; ${\upsigma}_{res,j}^{2}$ is the RT residual variance; ${\upsigma}_{\tau }^{2}$ is the variance of the speed parameters; ${\uprho}_{\theta \tau }$ is the correlation between the ability and speed parameters; ω is the regression weight of the ability parameter on the adaptability parameter; ${\upeta}_{0}$ and ${\upeta}_{1}$ are the location and scale parameters, respectively; ${q}_{j}$ is the latent item indicator variable; and ${C}_{ij}$ is the latent class variable of a person’s response to an item. The positive-dependency items were more prevalent than the negative-dependency items (3:1)

At the bottom of the tables, the CCRs for the item-level latent dependency indicator ${q}_{j}$ and the response-level latent variable ${C}_{ij}$ are reported. The results first showed that the dependency type of each item could be accurately identified, as indicated by the consistently high CCR values for ${q}_{j}$ across all conditions. This finding suggests that the proposed model can reliably recover the direction of local dependency at the item level.

Turning to the classification of response pace, the CCR for the response-level latent variable ${C}_{ij}$ reflected the model’s ability to correctly distinguish between faster- and slower-response processes. This classification performance was inherently linked to the recovery of the hierarchical mixing mechanism. Specifically, the person-level mixing probability was determined by the adaptability parameter, which was modeled as a function of the latent trait θ with regression coefficient ω, and subsequently linked to class membership through the regression parameters ${\upeta}_{0}$ and ${\upeta}_{1}$. Therefore, accurate estimation of ${\upeta}_{0}$, ${\upeta}_{1}$, and ω led to improved estimation of person-level mixing probabilities, which in turn enhanced the correctness of response-level class assignments.

Across the balanced dependency, negative-dominant, and positive-dominant conditions, the results showed that the RMSEs of the regression parameters ${\upeta}_{0}$ and ${\upeta}_{1}$, as well as the regression coefficient ω linking θ to the adaptability parameter, decreased as sample size and test length increased, while the recovery of θ improved with longer tests (as further discussed below). Taken together, more accurate estimation of θ and ω led to improved recovery of the adaptability parameter, which, combined with accurate estimation of ${\upeta}_{0}$ and ${\upeta}_{1}$, resulted in more precise estimation of person-level mixing probabilities.

This, in turn, corresponded to a clear increase in the CCR for response-level classification, with values generally ranging from approximately 0.81 to 0.83 across conditions. These CCR values indicate satisfactory and practically acceptable classification performance, particularly under conditions with larger sample sizes and longer tests. With respect to dependency composition, CCR was consistently slightly higher under the balanced condition than under the negative- and positive-dominant conditions, although the differences were small.

**Fig. 1:**
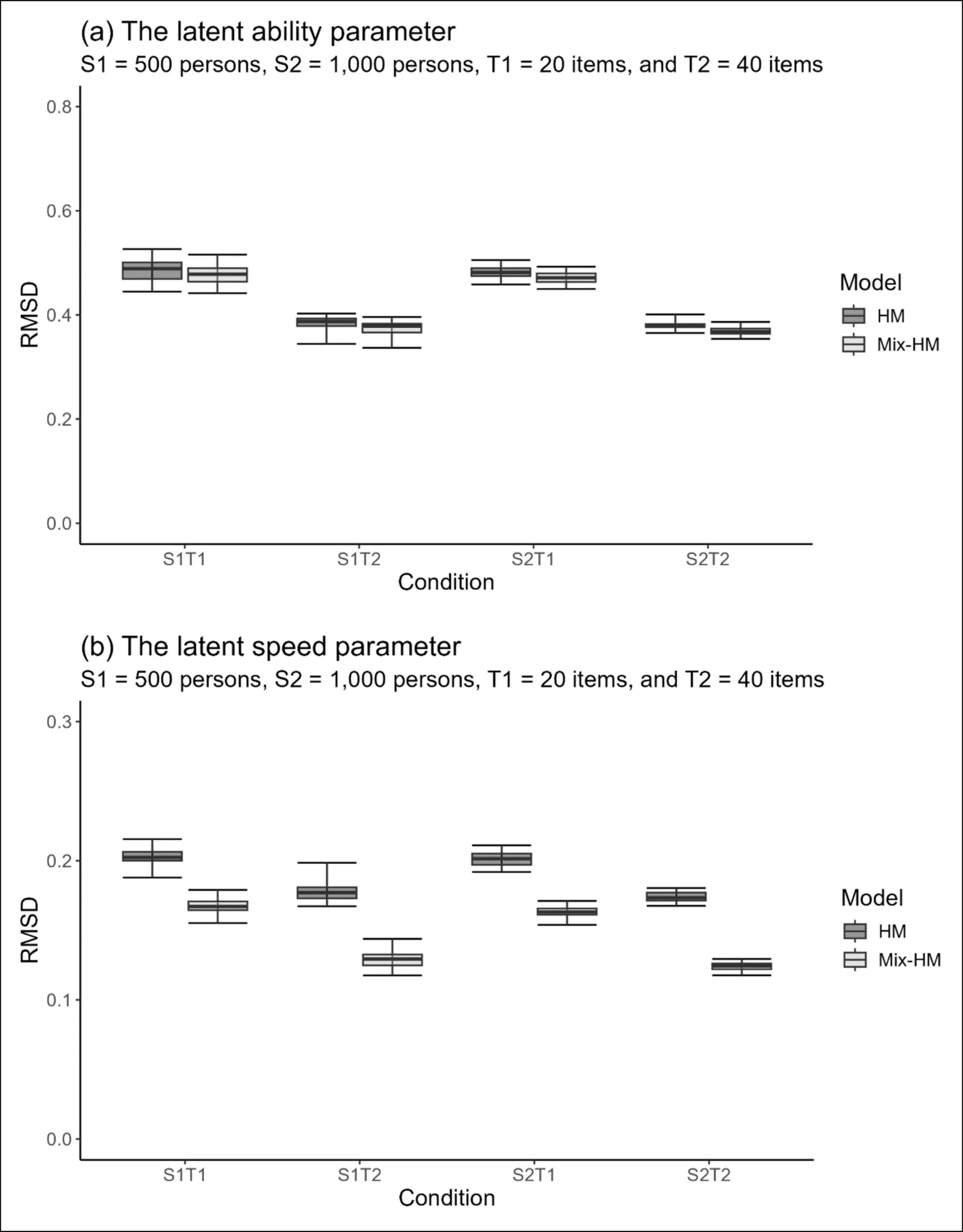
Box plots of RMSDs of person parameter estimates under the balanced dependency for the Mix-HM and the HM when data were generated from the Mix-HM. *Note*. HM = hierarchical model; Mix-HM = mixture hierarchical model. Positive- and negative-dependency items were equally distributed (1:1)

**Fig. 2:**
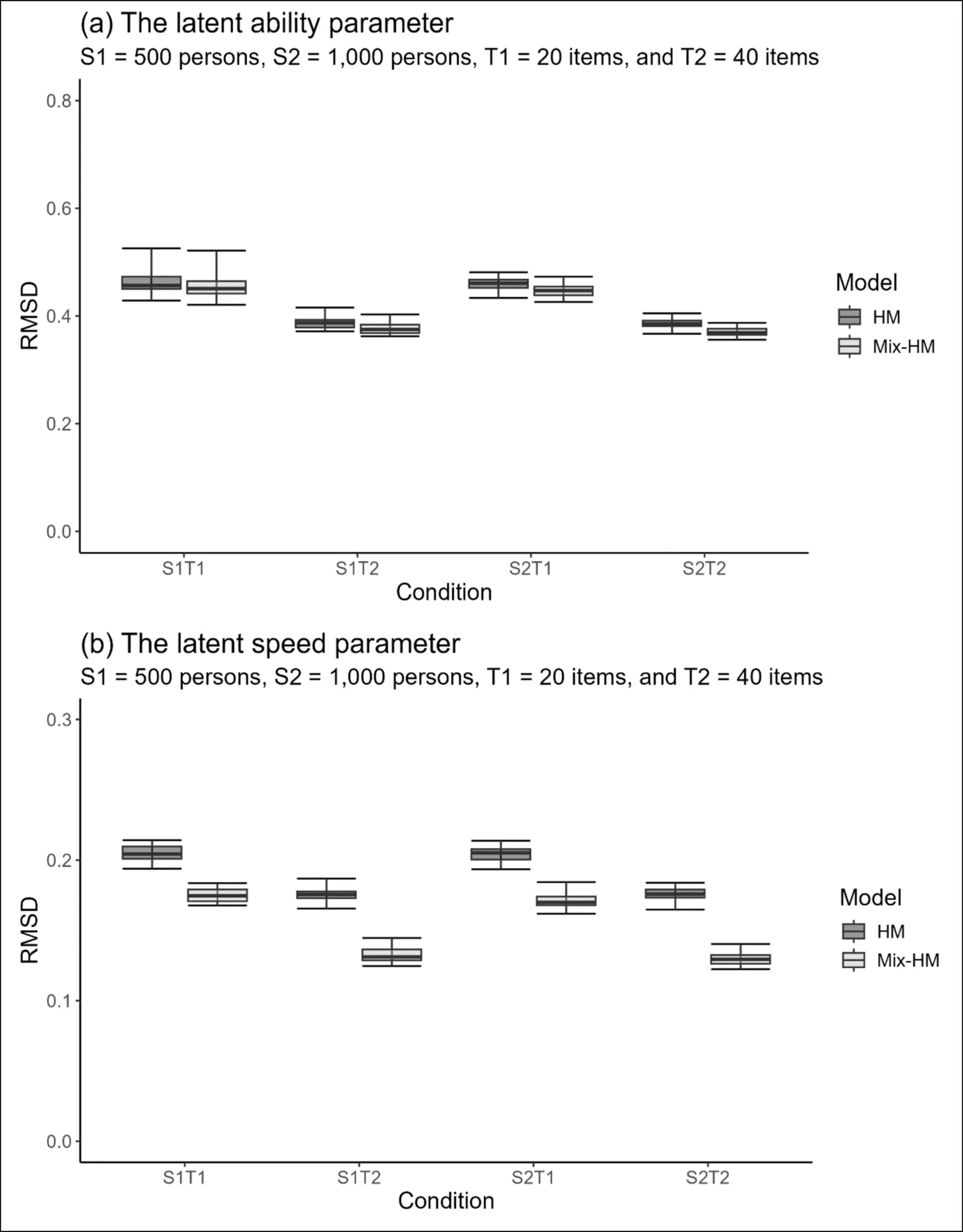
Box plots of RMSDs of person parameter estimates under the negative-dominant dependency for the Mix-HM and the HM when data were generated from the Mix-HM. *Note*. HM = hierarchical model; Mix-HM = mixture hierarchical model. Negative-dependency items were more prevalent than positive-dependency items (3:1)

**Fig. 3:**
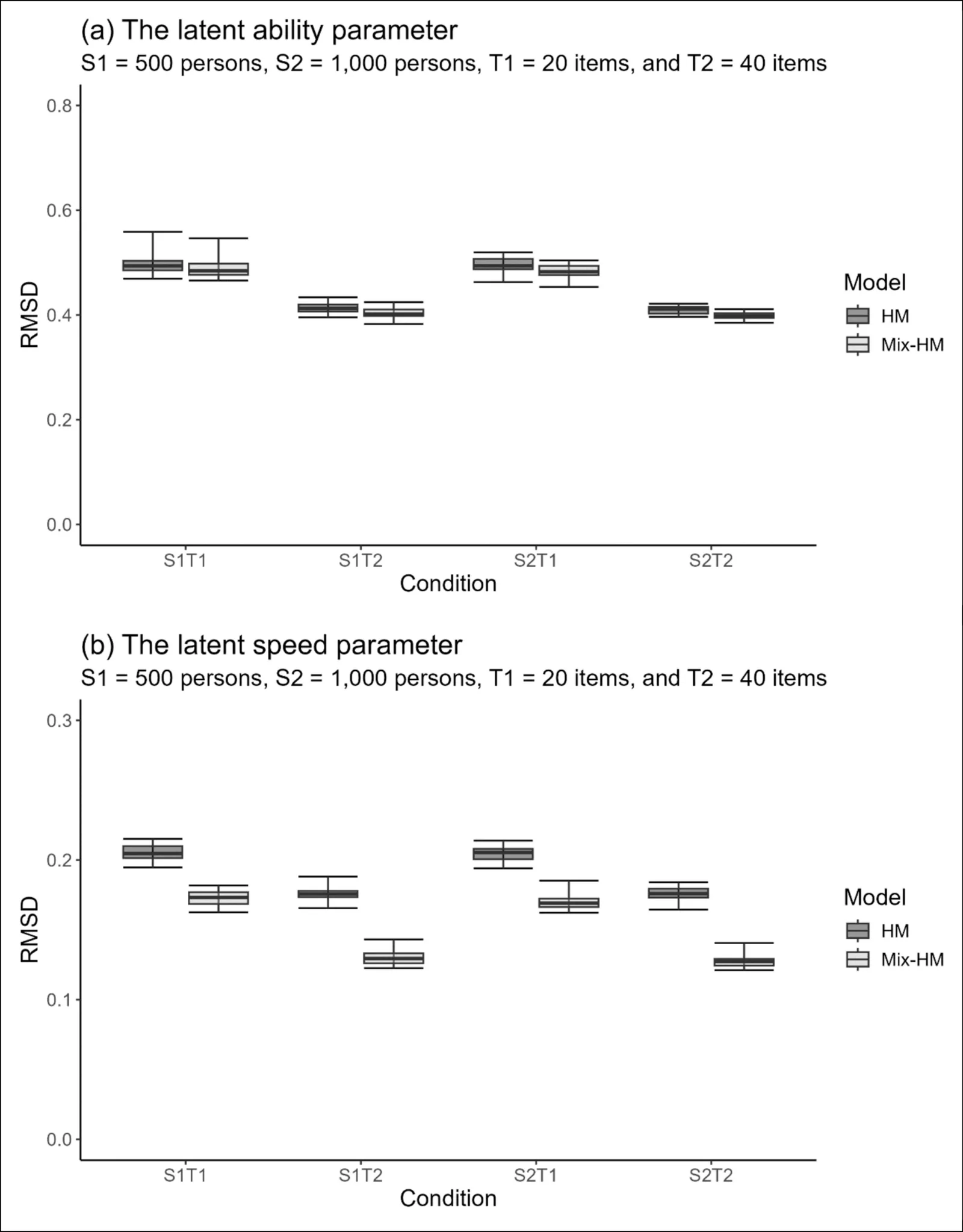
Box plots of RMSDs of person parameter estimates under the positive-dominant dependency for the Mix-HM and the HM when data were generated from the Mix-HM. *Note*. HM = hierarchical model; Mix-HM = mixture hierarchical model. Positive-dependency items were more prevalent than negative-dependency items (3:1)

Person parameter recovery was evaluated to assess the impact of ignoring local dependency between response accuracy and RT induced by different latent response classes. This evaluation was conducted by comparing the ability and speed parameter estimates obtained from the proposed Mix-HM with those derived from the conventional HM. Figures 1, 2, and 3 present box plots of RMSDs for person parameter estimates across replications under the balanced, negative-dependency–dominant, and positive-dependency–dominant conditions, respectively.

As expected, the Mix-HM consistently yielded lower RMSDs for the speed parameters than the HM did across all conditions, with the differences becoming more pronounced for longer tests. In contrast, the differences between the two models in terms of recovering the ability parameters were relatively small across all three dependency conditions, although the Mix-HM tended to yield slightly lower RMSDs. Similar patterns were observed across the balanced, negative-dominant, and positive-dominant dependency conditions.

Taken together, these results indicate that the advantage of the Mix-HM over the HM is more pronounced for speed parameter recovery than for ability parameter recovery. The relatively small differences in ability RMSDs may suggest that ability estimates are less sensitive to the forms of model misspecification considered in this study, although a further investigation is needed to clarify the underlying mechanisms.

## Simulation 2: Efficiency of the model fit criteria for model selection

### Design

In recent applied research conducted using Bayesian estimation, two model evaluation criteria—leave-one-out cross-validation with Pareto-smoothed importance sampling (PSIS-LOO-CV) and the widely applicable information criterion (WAIC) (Vehtari et al., 2017, 2019)—have been widely recommended for model comparisons and have been commonly used to select the best-fitting model based on Markov chain Monte Carlo (MCMC) sampling, particularly in mixture modeling contexts (e.g., Jin et al., 2022). Although these criteria have been shown to perform well in terms of distinguishing the true (or appropriate) model from misspecified alternatives, with relatively low false positive rates, their effectiveness for model selection in the context of the proposed Mix-HM remains unclear and warrants a systematic investigation through a simulation.

The focus of Simulation 2 was on evaluating the ability of the examined model fit criteria to correctly identify the true data-generating model among the Mix-HM and its reduced variants. To this end, several data-generating models were considered. These included the full Mix-HM, the Mix-HM with an invariance constraint on discrimination (Mix-HM-ICD), and the Mix-HM with an invariance constraint on difficulty (Mix-HM-ICDF), where the discrimination and difficulty parameters, respectively, were constrained to be equal across latent classes.

To provide a broader comparison, data were also generated from the conventional HM and a continuous-variable extension of the HM (CV-HM; Bolsinova et al., 2017a) for modeling conditional dependency between response accuracy and RT. The set of fitted models included all the aforementioned data-generating models, along with two additional constrained models: the Mix-HM with identical discrimination (Mix-HM-ID), obtained by fixing all discrimination parameters to 1, and the HM with identical discrimination (HM-ID). These two models were included only as candidate fitting models and were not used as data-generating models.

To align the simulation settings with the empirical analysis presented later, the sample size and test length were fixed at 500 examinees and 20 items, respectively. To assess the stability of the model fit criteria, the number of replications for each condition was increased to 100. This design was intended to enhance the reliability of the obtained simulation results. Under these settings, it was expected that both WAIC and PSIS-LOO-CV would demonstrate adequate power for correctly selecting the true model while avoiding the selection of misspecified alternatives.

### Results

Table 4 presents the model selection rates determined based on WAIC and PSIS-LOO-CV under different data-generating models. The values in each cell represent the proportions of selections across 100 replications, with bold values indicating the selection rates of the true model. In general, the results showed that most true models could be correctly identified, as their selection rates were consistently higher than those of the misspecified alternatives. This pattern suggests that both WAIC and PSIS-LOO-CV demonstrated adequate power for selecting the true model. However, for certain conditions, the hit rates were lower, warranting further examination.

**Table 4:** Model selection rates under different data-generating models in Simulation 2

		Model evaluation criterion	
True model	Fitted model	WAIC	PSIS-LOOCV
Mix-HM	HM	0.00	0.00
	HM-ID	0.00	0.00
	Mix-HM	**0.68**	**0.70**
	Mix-HM-ICD	0.28	0.26
	Mix-HM-ICDF	0.04	0.04
	Mix-HM-ID	0.00	0.00
	CV-HM	0.00	0.00
Mix-HM-ICD	HM	0.00	0.00
	HM-ID	0.00	0.00
	Mix-HM	0.06	0.16
	Mix-HM-ICD	**0.90**	**0.80**
	Mix-HM-ICDF	0.04	0.04
	Mix-HM-ID	0.00	0.00
	CV-HM	0.00	0.00
Mix-HM-ICDF	HM	0.00	0.00
	HM-ID	0.00	0.00
	Mix-HM	0.00	0.18
	Mix-HM-ICD	0.20	0.10
	Mix-HM-ICDF	**0.80**	**0.72**
	Mix-HM-ID	0.00	0.00
	CV-HM	0.00	0.00
HM	HM	**0.58**	**0.56**
	HM-ID	0.00	0.00
	Mix-HM	0.12	0.14
	Mix-HM-ICD	0.22	0.22
	Mix-HM-ICDF	0.08	0.08
	Mix-HM-ID	0.00	0.00
	CV-HM	0.00	0.00
CV-HM	HM	0.00	0.00
	HM-ID	0.00	0.00
	Mix-HM	0.00	0.00
	Mix-HM-ICD	0.00	0.00
	Mix-HM-ICDF	0.00	0.00
	Mix-HM-ID	0.02	0.02
	CV-HM	**0.98**	**0.98**

HM = hierarchical model; HM-ID = HM with identical discrimination; Mix-HM = mixture HM; Mix-HM-ICD = Mix-HM with an invariance constraint on discrimination; Mix-HM-ICDF = Mix-HM with an invariance constraint on difficulty; Mix-HM-ID = Mix-HM with identical discrimination; CV-HM = continuous-variable HM. The sample size and test length were fixed at 500 persons and 20 items, respectively. All other simulation conditions were identical to those in Simulation 1. The results were based on 100 replicated datasets. Bold numbers indicate the selection rates for the true (data-generating) model

First, when the true model was the Mix-HM, although the Mix-HM was the most frequently selected model, the Mix-HM-ICD also exhibited nonnegligible false-positive rates (0.28 for WAIC and 0.26 for PSIS-LOO-CV). This result suggests that the model imposing invariance on the discrimination parameters across the two speed classes could also provide a reasonable fit for the data. One possible explanation for this pattern relates to the magnitudes of the discrimination differences that were specified in the simulation design. Specifically, the discrimination difference parameter ${\updelta}_{j}^{\upalpha }$ was set to 1.5, while the discrimination parameters for the slower-response favoring class ranged from 0.5 to 1.5. Under this setting, the resulting discrimination parameters for the faster-response favoring class ranged approximately from 0.33 to 2.25, depending on the direction of dependency. Such differences, although present, may not be sufficiently large to provide strong discriminative information for distinguishing between the Mix-HM and Mix-HM-ICD.

To further examine this explanation, an additional simulation was conducted in which the true model remained the Mix-HM while the magnitude of the discrimination difference parameter ${\updelta}_{j}^{\upalpha }$ was increased to 2 (a moderate difference) and 2.5 (a large difference). As shown in Table 5, increasing ${\updelta}_{j}^{\upalpha }$​ led to a substantial improvement in the correct selection rate of the Mix-HM. Specifically, the hit rates increased to above 0.90 across conditions, and they were close to 1.00 when ${\updelta}_{j}^{\upalpha }=2.5$. These results suggest that larger discrimination differences provide clearer information for distinguishing between the Mix-HM and its constrained variants, thereby improving the model selection performance.

**Table 5:** Model selection rates when the data-generating model was the Mix-HM under varying discrimination difference conditions in Simulation 2

		Model evaluation criterion	
${\updelta }^{\upalpha }$	Fitted model	WAIC	PSIS-LOOCV
2	HM	0.00	0.00
	HM-ID	0.00	0.00
	Mix-HM	**0.90**	**0.90**
	Mix-HM-ICD	0.08	0.10
	Mix-HM-ICDF	0.02	0.00
	Mix-HM-ID	0.00	0.00
	CV-HM	0.00	0.00
2.5	HM	0.00	0.00
	HM-ID	0.00	0.00
	Mix-HM	**0.98**	**0.98**
	Mix-HM-ICD	0.02	0.02
	Mix-HM-ICDF	0.00	0.00
	Mix-HM-ID	0.00	0.00
	CV-HM	0.00	0.00

HM = hierarchical model; HM-ID = HM with identical discrimination; Mix-HM = mixture HM; Mix-HM-ICD = Mix-HM with an invariance constraint on discrimination; Mix-HM-ICDF = Mix-HM with an invariance constraint on difficulty; Mix-HM-ID = Mix-HM with identical discrimination; CV-HM = continuous-variable HM. The sample size and test length were fixed at 500 persons and 20 items, respectively. The discrimination difference parameter ${\updelta }^{\upalpha }$ varied across the simulation conditions. The results were based on 100 replicated datasets. Bold numbers indicate the selection rates for the true (data-generating) model

Next, when the true model was the HM, the proportion of times that the HM was correctly selected as the best-fitting model was relatively modest. This finding suggests that both WAIC and PSIS-LOO-CV may fail to impose sufficient penalties on the model complexity level, thereby leading to a tendency to favor more complex models. Similar patterns have been reported in previous studies, where information-based criteria may prefer over-parameterized models under certain conditions (e.g., Li et al., 2009). Therefore, in addition to relying on model fit criteria, it is important to further examine the estimated parameters within complex models (i.e., the Mix-HM) to assess whether the additional imposed complexity is substantively justified.

Building on the results reported in Table 4, where the Mix-HM was repeatedly fitted to HM-generated data across 100 replications and evaluated using WAIC and PSIS-LOO-CV, we further examined the corresponding parameter estimates to assess whether the selected model was substantively supported. In particular, attention was given to the item-level dependency indicators ${q}_{j}$​. Under this scenario, where the true model did not involve dependency structures, the ${q}_{j}$​ indicators were not expected to exhibit systematic patterns. Accordingly, their posterior probabilities should be close to 0.5, reflecting random fluctuations between the two latent states.

**Fig. 4:**
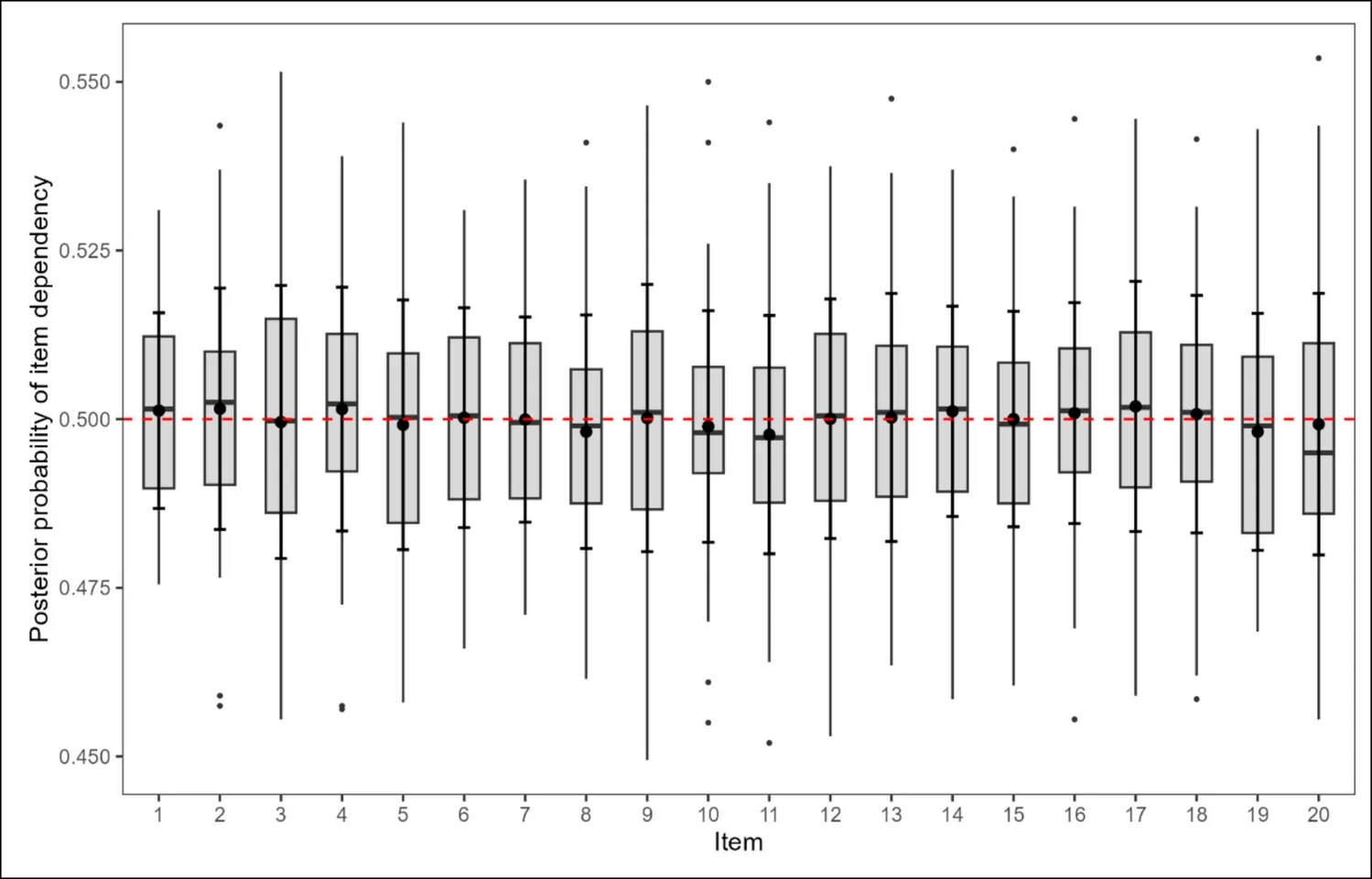
Posterior probabilities of item-level dependency indicators when fitting the Mix-HM to HM-generated data. *Note.* Posterior probabilities were computed as the posterior means of binary item-level dependency indicators (${q}_{j}$), distinguishing positive versus negative local dependence. Each boxplot summarizes 100 replications, and error bars represent ± 1 standard deviation. The dashed line at 0.5 indicates the absence of an identifiable dependency direction. The sample size and test length were fixed at 500 persons and 20 items, respectively

As shown in Fig. 4, the box plots of the posterior probabilities of ${q}_{j}$​ determined across replications indicate that the estimates produced for all items were consistently centered around 0.5. This result suggests that, despite occasionally favoring the Mix-HM based on model fit criteria, the estimated dependency structure lacked substantive support when the data were generated from the HM.

## Empirical demonstration

To demonstrate the application of the Mix-HM to real data, we used the computer-based version of the Programme for the International Assessment of Adult Competencies (PIAAC), an international large-scale assessment. The PIAAC employs a multistage computerized adaptive approach to present test items on the basis of booklets and covers four major content areas: literacy, numeracy, reading components, and problem solving. The first cycle of the PIAAC administration spanned from 2012 to 2018 and included 38 countries. For our empirical example, we focused on English-speaking participants who took the numeracy test. We selected a combination of two booklets, where the first-stage and second-stage booklets were of low and intermediate difficulty, respectively. This selection ensured a sufficient number of samples and followed the procedure used in a previous study by Nagy and Ulitzsch (2022). After respondents with missing responses and RTs were excluded, the dataset comprised responses from 651 individuals to 20 items, which were used for the data analysis.

**Fig. 5:**
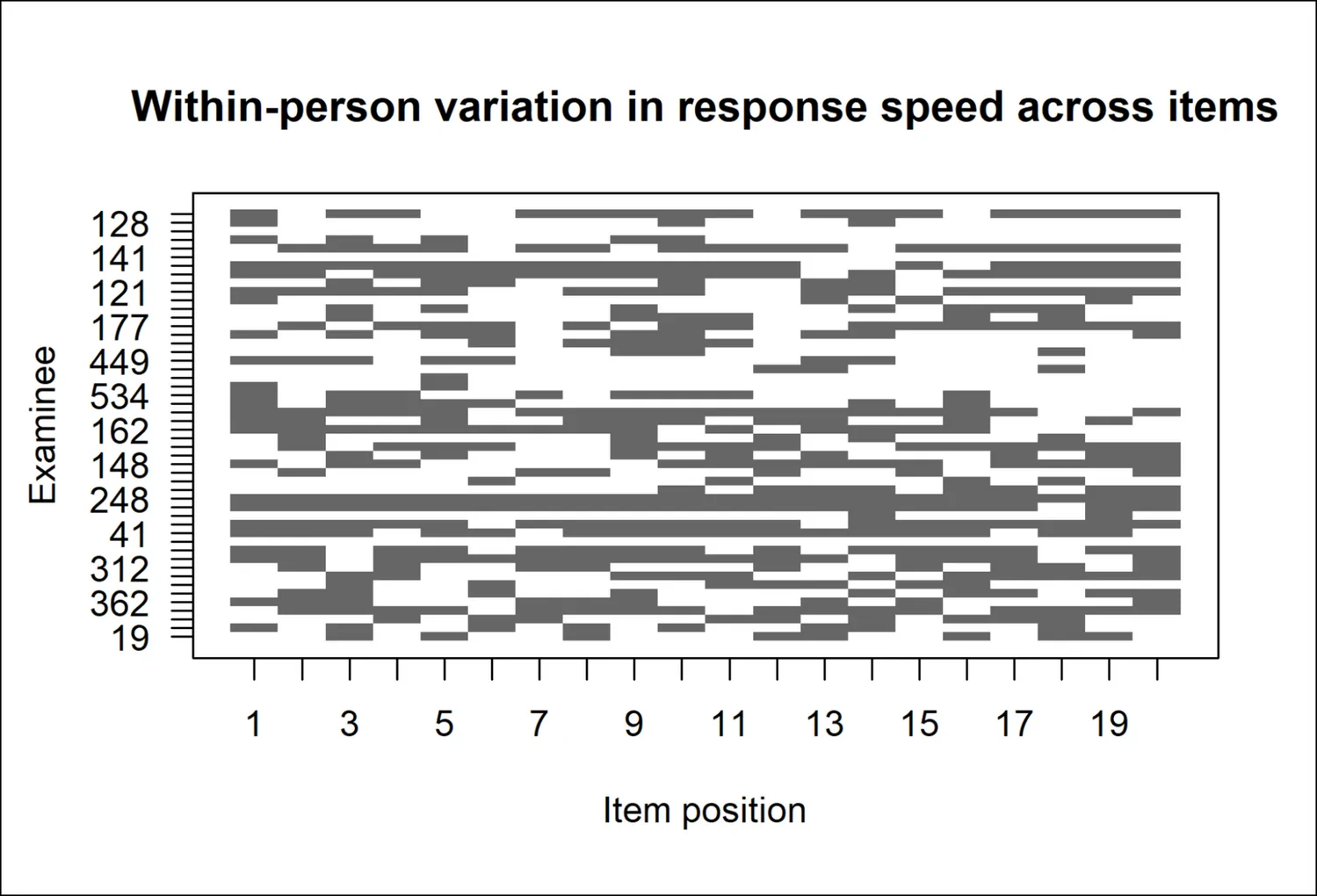
Within-person variation in response speed across items in the PIAAC numeracy assessment. *Note.* Rows represent 50 randomly selected examinees, and columns represent item positions. Gray cells indicate RTs longer than the item median (slow), whereas white cells indicate response times shorter than the item median (fast)

**Fig. 6:**
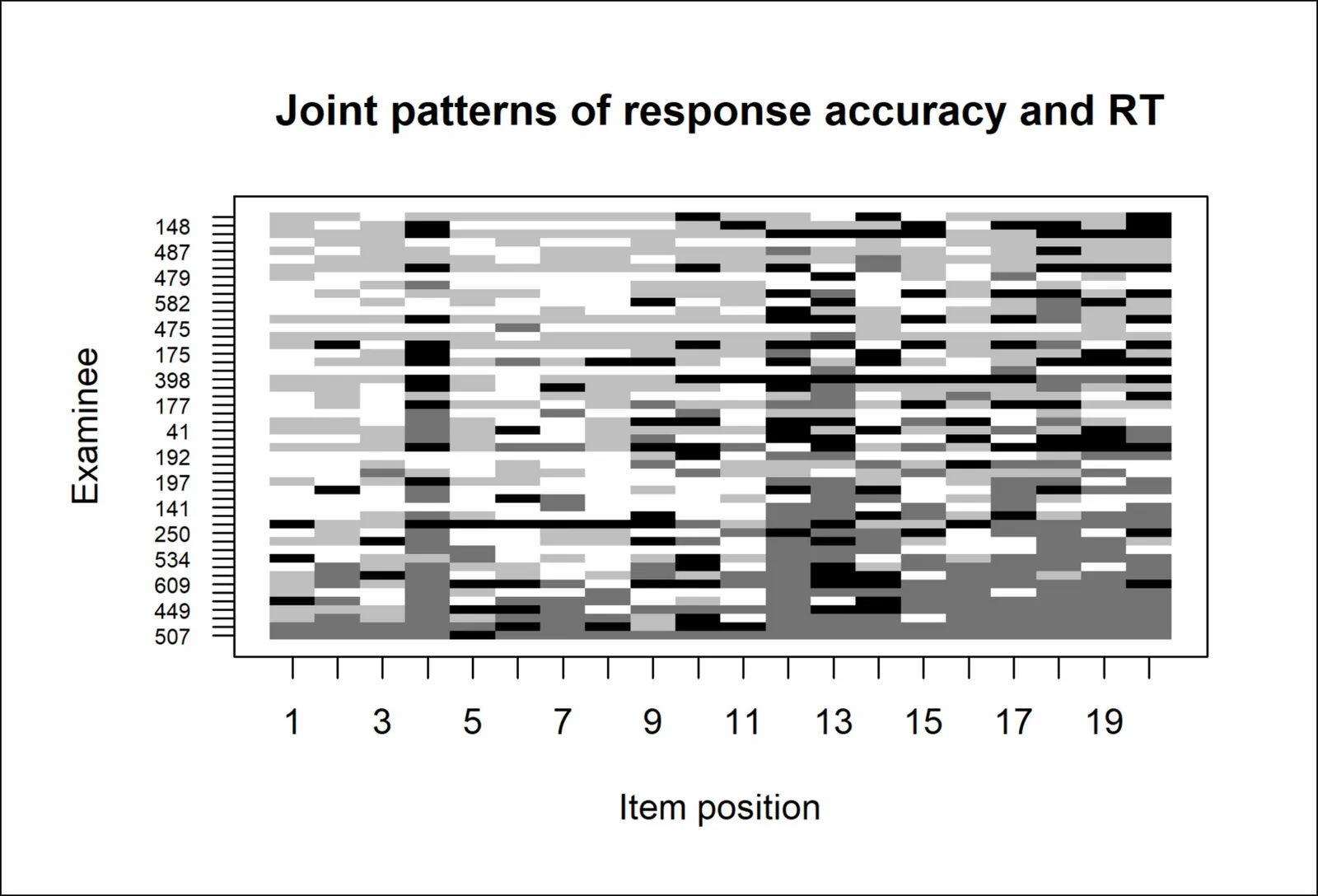
Joint patterns of response accuracy and RT across items in the PIAAC numeracy assessment. *Note.* Rows represent 50 randomly selected examinees and columns represent item positions. RTs were classified relative to the item-specific median. White cells denote fast and correct responses, light gray cells denote slow and correct responses, dark gray cells denote fast and incorrect responses, and black cells denote slow and incorrect responses

To provide preliminary descriptive evidence before applying the proposed model, we conducted a simple visual exploratory analysis of RT and accuracy patterns. We randomly selected 50 examinees and inspected their response behaviors across items. Figure 5 illustrates the within-person response speed variations observed across different item positions, where the RTs were classified relative to the item-specific median. The figure shows that many examinees alternated between slower and faster responses across various items rather than responding at a fixed pace. Figure 6 further displays the joint patterns of response accuracy and RT by classifying the responses into four categories (fast–correct, slow–correct, fast–incorrect, and slow–incorrect). The patterns suggest that the relationship between speed and accuracy is heterogeneous: fast responses are not always associated with incorrect answers, and slow responses are not consistently linked to higher accuracy. Although these visualizations provide descriptive insights into the variability in response processes, a statistical measurement model is required to formally examine potential dependencies between accuracy and RT, particularly to account for within-person heterogeneity in response processes that cannot be adequately captured by descriptive analyses alone.

Before fitting the proposed Mix-HM to the data, it is crucial to consider the severity of local dependencies between responses and RTs and determine whether a complex model is necessary over a simpler hierarchical model. Within the Bayesian estimation framework, applying posterior predictive model checking (PPMC) methods to assess the absolute model–data fit is a convenient and efficient approach for detecting systematic discrepancies by comparing observed and replicated data over many iterations via a chosen statistical measure (Gelman et al., 1996).

The chosen statistical measure was the correlation coefficient between ability and residual log-transformed RT at the item level, following the approach proposed by Bolsinova and Tijmstra (2018) to detect potential conditional dependencies in an HM. The difference in the correlation coefficient between the observed and replicated data was then calculated for each item. The proportion of iterations in which the replicated correlation was larger than the observed correlation served as an indicator to evaluate whether the data were appropriately captured by the fitted model. An extremely large proportion (close to zero or 1) indicates that the log-transformed RT cannot be explained by speed, suggesting that local dependencies may occur and impact the data when an HM is applied.

**Fig. 7:**
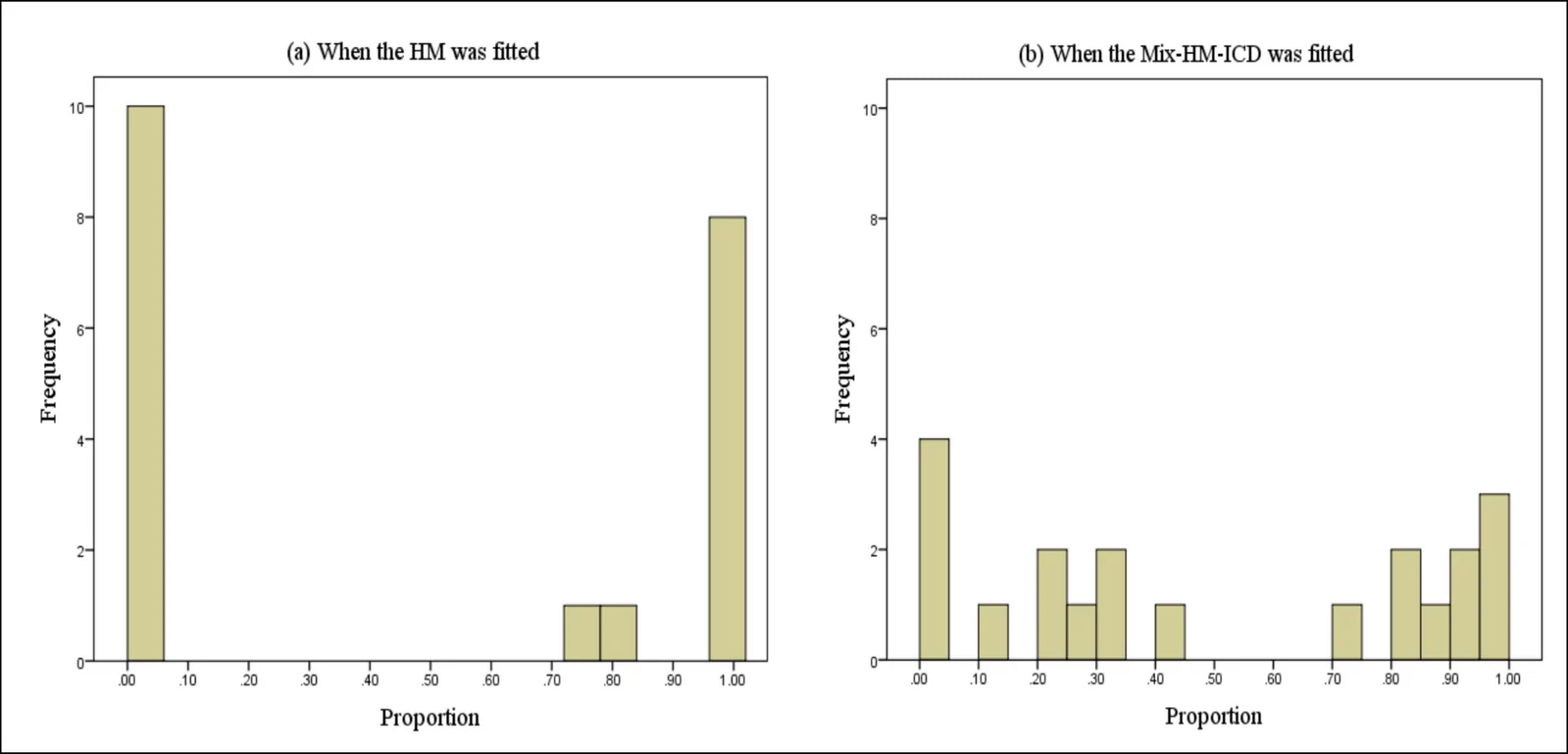
Results of the posterior predictive model checking for local dependencies on the basis of the correlation between ability and residual log-transformed RT. *Note*. HM = hierarchical model; Mix-HM-ICD = mixture hierarchical model with an invariance constraint on discrimination

Figure 7a shows a histogram of the PPMC proportions yielded across different items for the conventional HM, which assumed conditional independence. The large number of items exhibiting extremely large proportions indicates that the HM failed to capture an important aspect of the data, suggesting that local dependencies may exist and justifying the use of a more complex model.

A set of competing models developed from three modeling perspectives—non-mixture, mixture-based, and the CV-HM framework—were fitted for comparison purposes. The non-mixture models included the conventional HM and its constrained version with identical discrimination (HM-ID). The mixture-based models consisted of the Mix-HM as the most general specification, along with its variants: Mix-HM-ICD, Mix-HM-ICDF, and Mix-HM-ID. In addition, a further extension, Mix-HM-FD, was included to relax the constraint on the direction of discrimination differences across speed classes, allowing the relative magnitudes of the discrimination parameters between classes to vary more flexibly.

For comparison, the CV-HM and its corresponding variants (CV-HM-ICD, CV-HM-ICDF, and CV-HM-ID), which were defined in parallel with the Mix-HM specifications, were also included. Model comparisons were conducted using WAIC and PSIS-LOO-CV (Vehtari et al., 2017, 2019), with lower values indicating better model fit.

Table 6 shows the results obtained from the two criteria for the 11 fitted models. As expected, test-takers responded at different paces to the administered items during the test, and their performance was significantly influenced by the different speed classes to which they were classified. Nevertheless, the most complex Mix-HM was not chosen as the best-fitting model; instead, its reduced version, Mix-HM-ICD, yielded the lowest PSIS- WAIC and LOOCV values, making it the best-fitting model for the given data.

**Table 6:** Comparison of the model fit for the numeracy test via the PIAAC dataset

Fitted model	WAIC	PSIS-LOOCV
Non-mixture models		
HM	30,255.3	30,273.0
HM-ID	30,329.3	30,341.7
Mixture-based models		
Mix-HM	28,780.2	28,883.9
Mix-HM-ICD	**28,747.2**	**28,828.6**
Mix-HM-ICDF	28,774.0	28,874.4
Mix-HM-FD	28,754.2	28,858.8
Mix-HM-ID	28,816.3	28,881.1
Continuous-based models		
CV-HM	30,025.0	30,059.4
CV-HM-ICD	30,096.9	30,114.8
CV-HM-ICDF	30,208.5	30,241.6
CV-HM-ID	30,132.0	30,145.7

HM = hierarchical model; HM-ID = HM with identical discrimination; Mix-HM = mixture HM; Mix-HM-ICD = Mix-HM with an invariance constraint on discrimination; Mix-HM-ICDF = Mix-HM with an invariance constraint on difficulty; Mix-HM-FD = Mix-HM with free discrimination difference; Mix-HM-ID = Mix-HM with identical discrimination; CV-HM = continuous-variable HM; CV-HM-ICD = CV-HM with an invariance constraint on discrimination; CV-HM-ICDF = CV-HM with an invariance constraint on difficulty; CV-HM-ID = CV-HM with identical discrimination. Numbers in bold indicate the best-fitting model

**Fig. 8:**
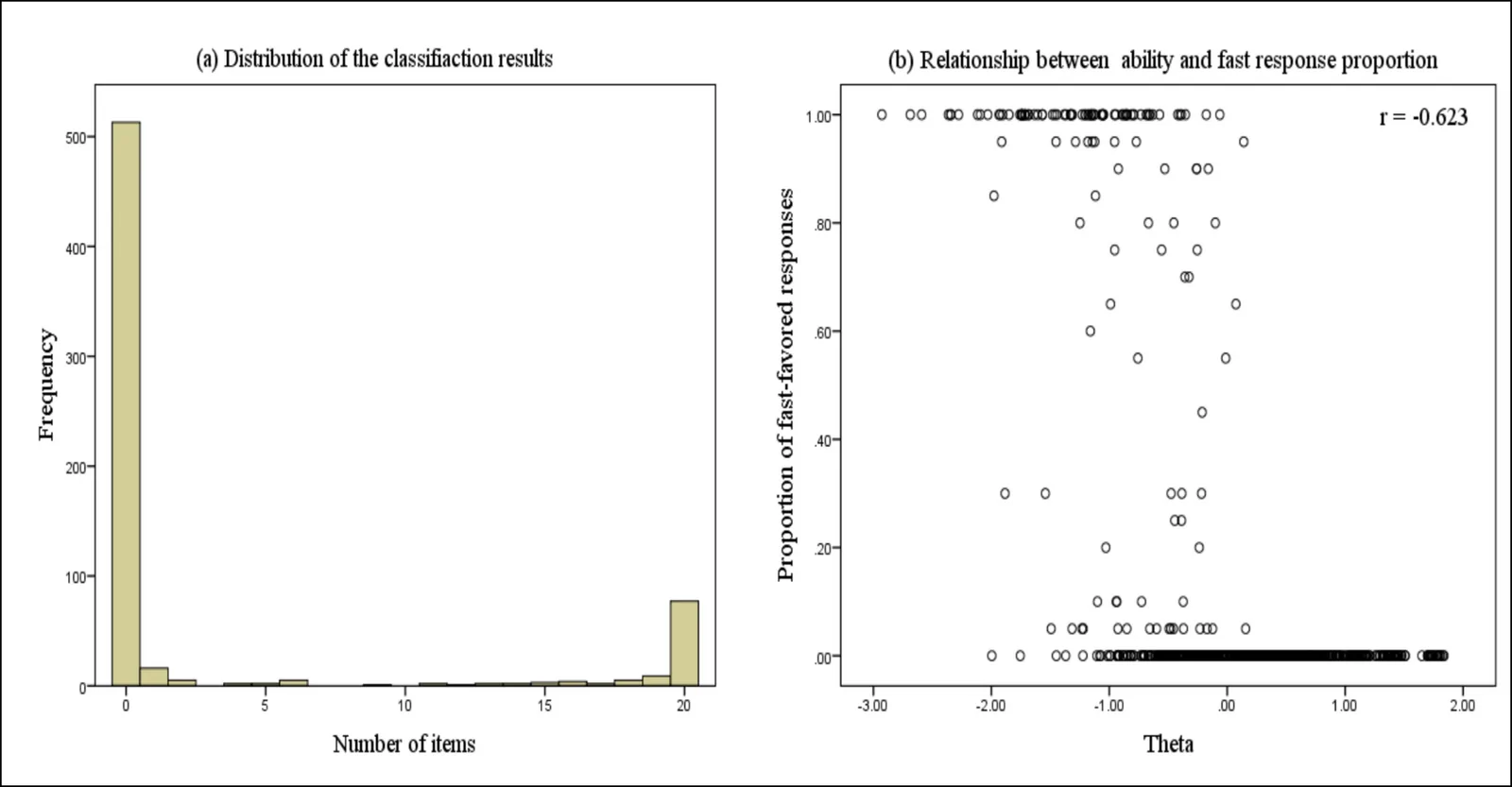
Results of fitting the Mix-HM-ICD to the numeracy test data. (**a**) Distribution of the number of items a person responds to at a faster pace. (**b**) Relationship between ability and faster response proportion. *Note.* Mix-HM-ICD = mixture hierarchical model with an invariance constraint on discrimination

Under the fit of Mix-HM-ICD, we first examined the response-speed favoring statuses exhibited by the test-takers throughout the test. Most test-takers responded to items at a consistent pace, as shown in Fig. 8a, with nearly 75% of the test-takers being classified as slower across all items. All items tended to exhibit positive dependency, suggesting that the slower-response favoring class may have derived greater benefit from the items than the faster-response favoring class did. Given that all items exhibited positive dependency (see the parameter estimates reported below), it is reasonable to infer that the more a test-taker tended to favor faster responses, the lower their estimated ability would be. Consistent with this expectation, the correlation between the proportion of individuals in the faster-favored class and the ability parameter estimate was − 0.63, as shown in the scatter plot presented in Fig. 8b.

**Fig. 9:**
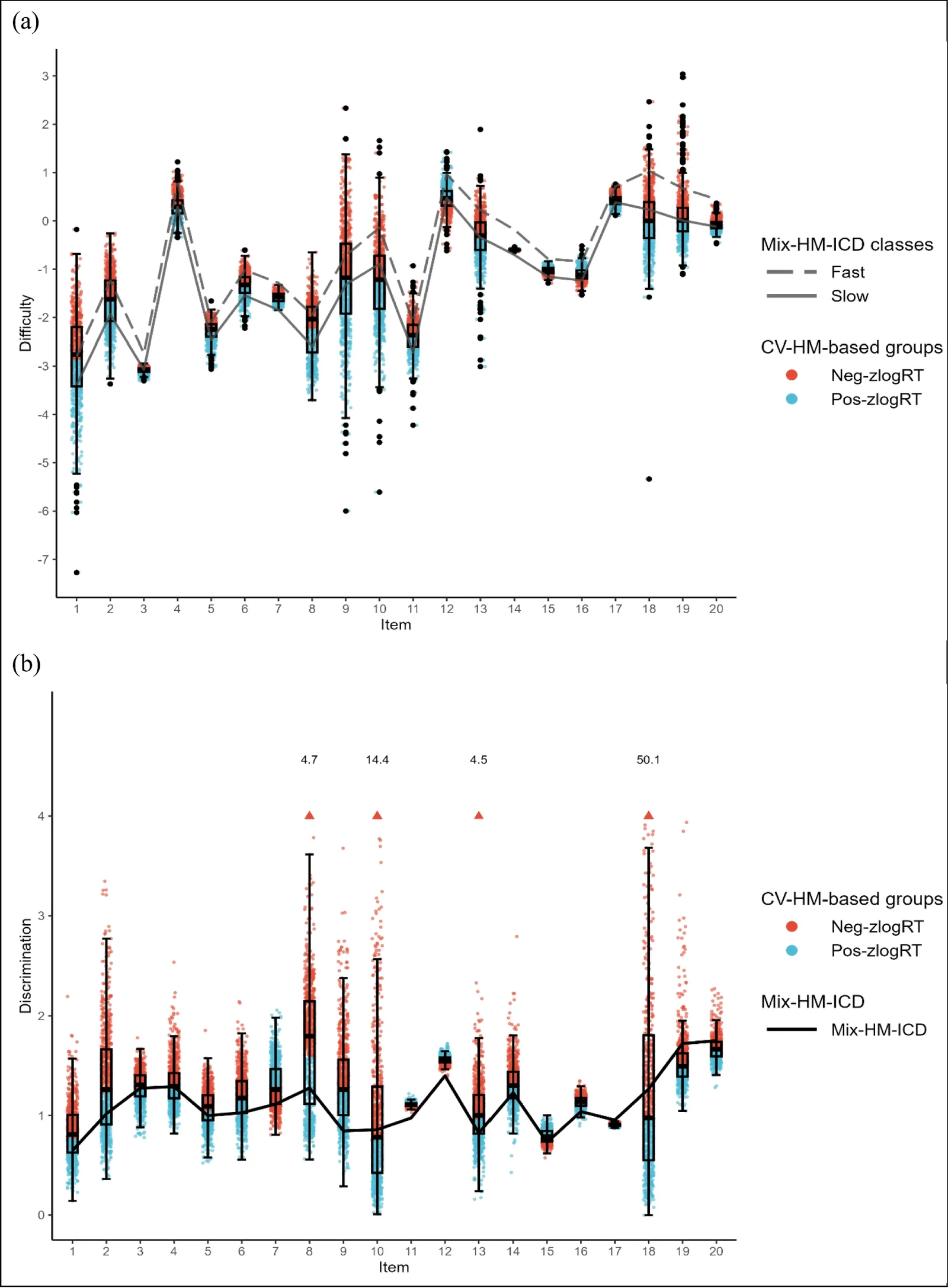
Comparison of item parameter estimates between the Mix-HM-ICD and the CV-HM for the PIAAC numeracy test data: (**a**) difficulty parameters and (**b**) discrimination parameters. *Note*. Mix-HM-ICD = mixture hierarchical model with an invariance constraint on discrimination; CV-HM = continuous-variable hierarchical model. The Mix-HM-ICD estimates are presented for the fast- and slow-response favoring latent classes. In the CV-HM, Neg-zlogRT and Pos-zlogRT denote individuals with negative and positive standardized log-response time residuals, respectively, corresponding to relatively faster and slower response behavior. In panel (**b**), triangles indicate discrimination estimates exceeding the upper limit of the *y*-axis (i.e., values greater than 4), and the numbers above the triangles denote the maximum estimated values for the corresponding items

To show the item parameter estimation differences between the best-fitting Mix-HM-ICD and the CV-HM, we summarized the parameter estimates yielded for the item difficulty and discrimination parameters using box-plot visualizations, as shown in Fig. 9a and b, respectively. Recall that because the item parameter estimates in the CV-HM completely depend on individual standardized log-response time residuals, each test-taker had distinct item parameter estimates, resulting in a continuous spread of values.

Focusing first on the item difficulty parameters, the Mix-HM-ICD produced two distinct estimates for each item corresponding to the two latent speed classes. Across all items, the difficulty parameters observed for the faster-response favoring class were consistently higher than those seen for the slower-response favoring class, indicating a clear positive dependency pattern between the response accuracy and RT. Specifically, the difficulty parameter estimates ranged from − 3.39 to 0.48 (*M* = − 1.20) for the slower-response favoring class and from − 2.80 to 1.04 (*M* = − 0.65) for the faster-response favoring class.

A similar pattern could be observed in the CV-HM results. For most items, individuals with negative standardized log-response time residuals (i.e., the faster-response group, Neg-zlogRT) tended to have higher difficulty parameter estimates than those with positive residuals (i.e., the slower-response group, Pos-zlogRT), again suggesting a general tendency toward positive dependency. However, unlike those of the Mix-HM-ICD, the CV-HM estimates exhibited substantially greater variability and dispersion. Because the CV-HM relies heavily on standardized log-response time residuals, the resulting difficulty estimates were highly sensitive to extreme values, which may have led to outliers and unstable parameter estimates.

As discussed earlier, this pattern may be explained by the fact that the CV-HM is less parsimonious and assumes that item parameter shifts are deterministic functions of standardized RT residuals, without accounting for potential random fluctuations. This characteristic may contribute to its relatively poorer model fit compared to that of the Mix-HM-ICD.

Turning to the discrimination parameters, as shown in Fig. 9b, the estimates obtained from the CV-HM exhibited even more pronounced variability and extreme values than those observed for the difficulty parameters. Due to the scale limitation of the *y*-axis in the figure, discrimination estimates exceeding 4 are represented by triangles, with the corresponding values labeled above each item. For instance, the maximum discrimination estimate for Item 18 reached 50.1, which clearly exceeded a plausible range for item discrimination parameters. These results further highlight the sensitivity of the CV-HM to extreme standardized log-response time residuals, leading to unstable and potentially unrealistic parameter estimates.

In contrast, the Mix-HM-ICD imposed a shared discrimination parameter across the two latent speed classes, resulting in substantially more stable estimates. The discrimination parameter estimates ranged from 0.65 to 1.75 (*M* = 1.11) across the items, which fell within a reasonable and interpretable range. Overall, these findings suggest that the Mix-HM-ICD can effectively mitigate the instability and extreme estimation issues observed in the CV-HM while providing a more parsimonious and interpretable item discrimination representation.

**Fig. 10:**
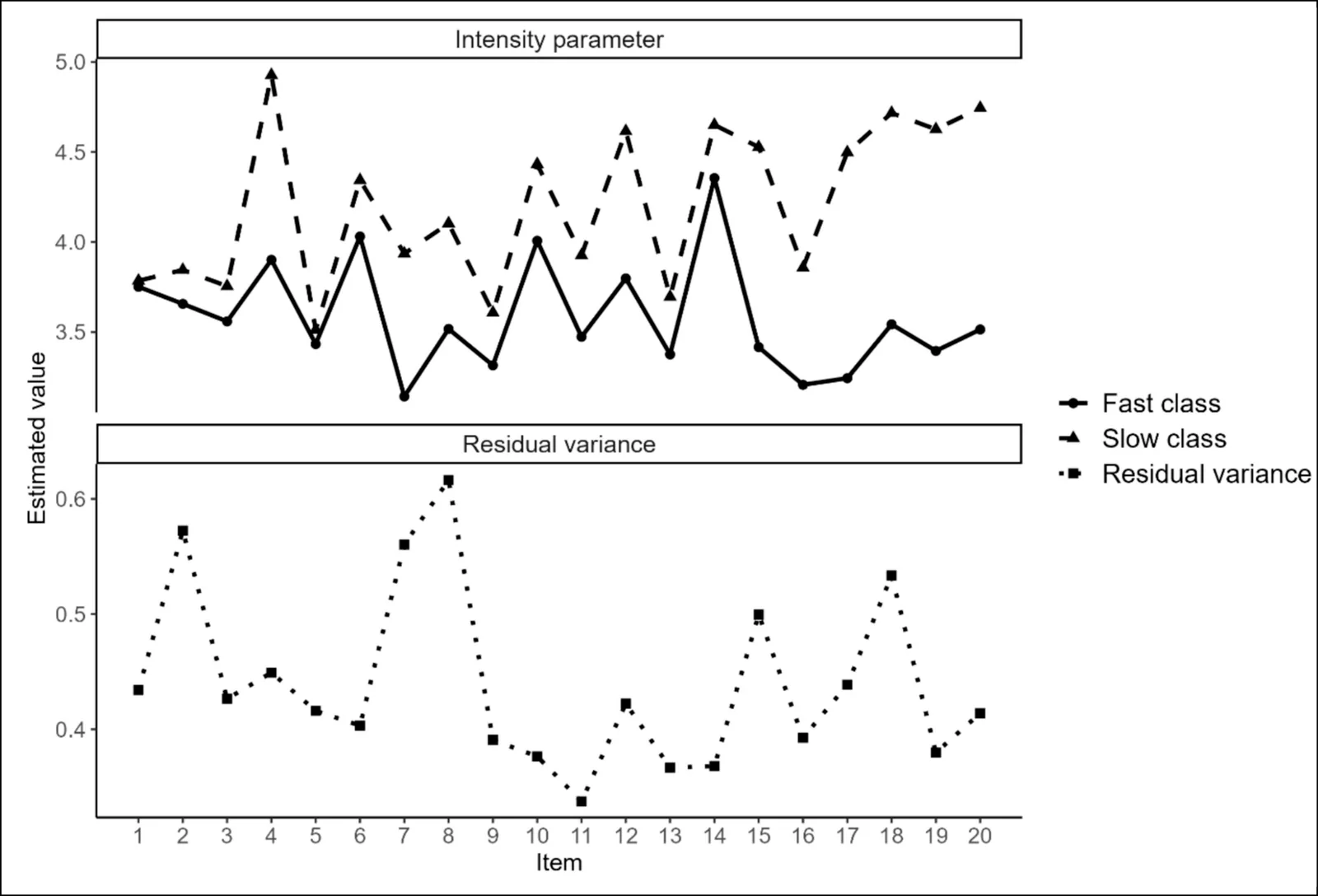
Item-level estimates of residual variance and intensity parameters across latent speed classes under the Mix-HM-ICD for the PIAAC numeracy test data. *Note.* Mix-HM-ICD = mixture hierarchical model with an invariance constraint on discrimination

In addition to the item difficulty and discrimination parameter estimates reported above, Fig. 10 presents the item-level estimates of the time intensity parameters and residual variances produced under the log-response time model fitted by the Mix-HM-ICD. The estimated time intensity parameters ranged from 3.51 to 4.93 (*M* = 4.21) for the slower-response favoring class and from 3.14 to 4.36 (*M* = 3.58) for the faster-response favoring class. The estimated residual variances ranged from 0.34 to 0.62 (*M* = 0.44). In addition, the variance of the latent speed parameter was estimated to be 0.12, and the correlation between the latent ability and speed parameters was 0.14. The estimated regression coefficient of ability on the propensity for faster responses (i.e., ω) was − 0.85, closely matching the value used in the simulation study.

**Fig. 11:**
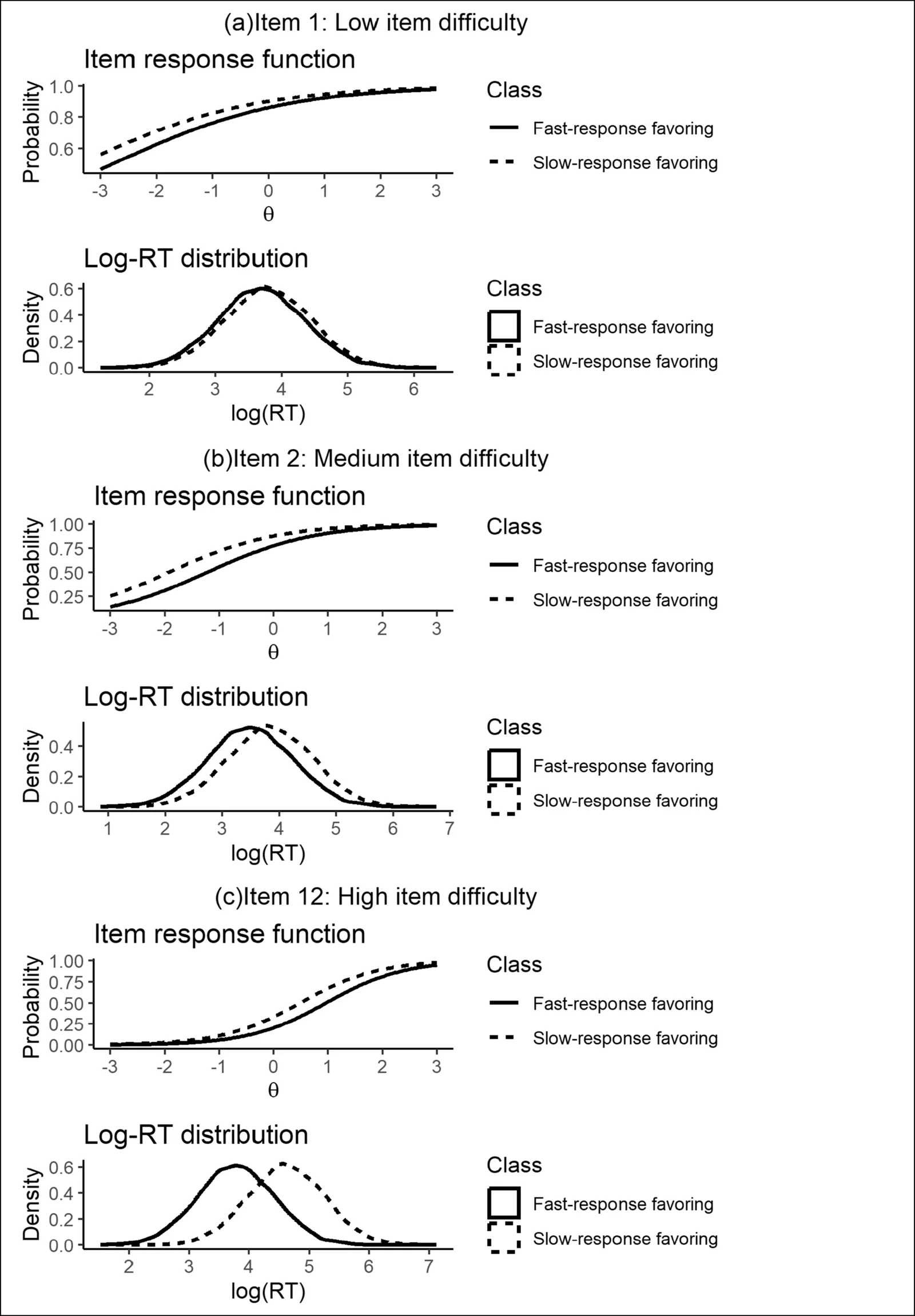
Examples of item response functions and log-response time distributions for three representative items in the PIAAC numeracy test

Three representative items were selected to illustrate their item response functions (IRFs) and log-RT distributions across different difficulty levels (low, medium, and high), as shown in Fig. 11. Panels a, b, and c correspond to Item 1 (low difficulty), Item 2 (medium difficulty), and Item 12 (high difficulty), respectively. The IRFs and log-RT distributions were constructed using parameter estimates derived from the fitted Mix-HM-ICD (see Figs. 9 and 10). For Item 1, the difference in response probabilities between the faster- and slower-response favoring classes was most evident in the lower ability range, reflecting a positive dependency pattern, whereas the log-RT distributions of the two classes were highly similar. Consistent with Bolsinova et al. (2017c), who suggested that differences in cautiousness contribute to positive conditional dependency, the present results further indicate that the RT differences between the fast and slow classes diminished when the item difficulty level was low. For Item 2, the difference in response probabilities became more pronounced near the middle range of ability, and the separation between the log-RT distributions increased, suggesting that the influence of cautiousness on RTs became more evident as the item difficulty level increased.

For Item 12, the difference in response probabilities between the two classes was most substantial in the higher ability range and accompanied by the greatest separation in the log-RT distributions. This pattern suggests that more difficult items may involve increased engagement of controlled cognitive processes, which in turn strengthens both the dependency between accuracy and RT and the divergence between latent speed classes (De Boeck & Jeon, 2019). Overall, these results demonstrate how item difficulty interacts with latent speed classes to jointly influence response accuracy and RT.

**Fig. 12:**
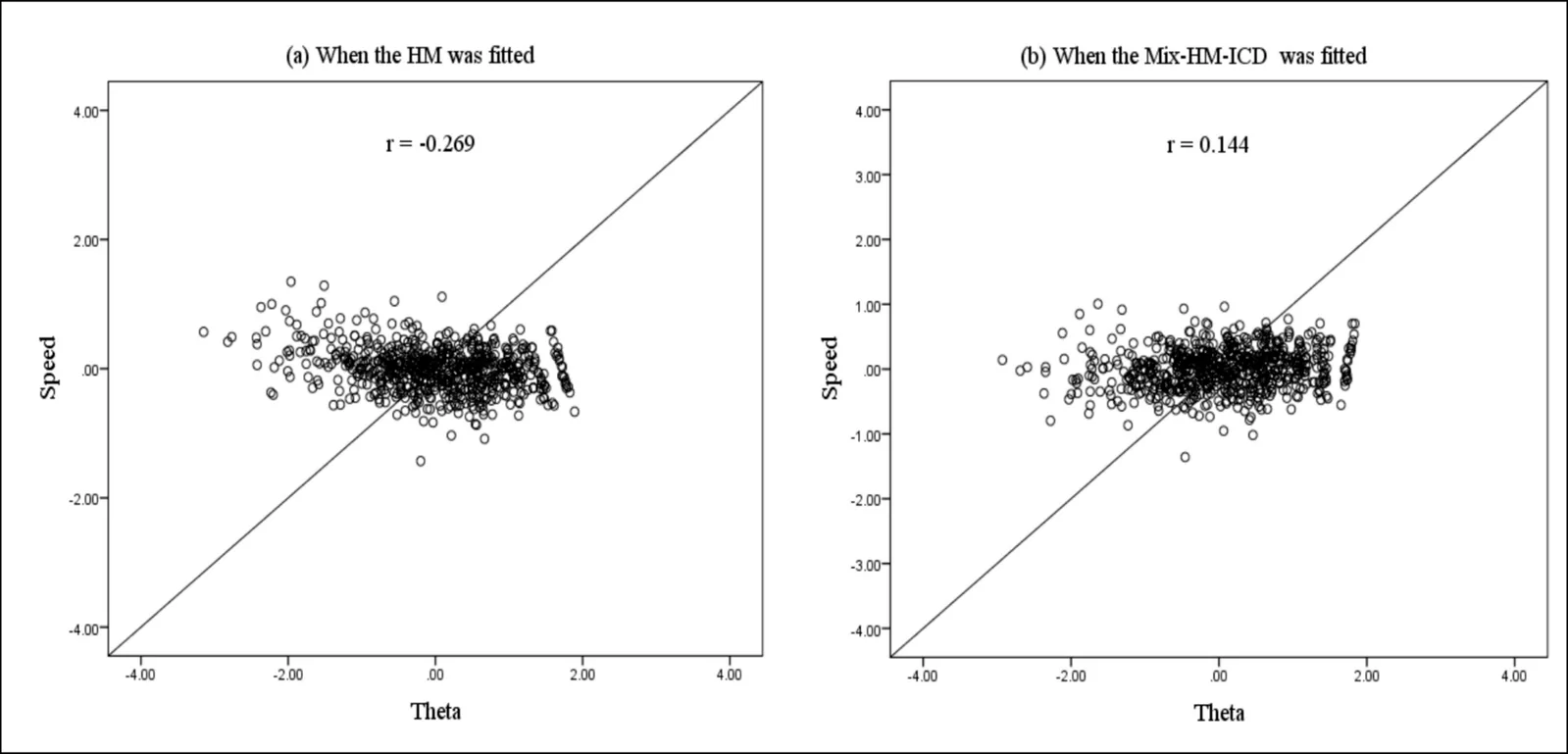
Relationship between the latent ability and speed parameter estimates under (**a**) the fit of the HM and (**b**) the best-fitting Mix-HM-ICD. *Note.* HM = hierarchical model; Mix-HM-ICD = mixture hierarchical model with an invariance constraint on discrimination

Researchers may raise an important question regarding whether ignoring potential mixtures of speed could lead to misleading inferences of individuals’ measures. To address this, we compared the correlation between the latent ability and speed parameter estimates using the best-fitting Mix-HM-ICD with that obtained from the conventional HM. Figure 12a and b display scatter plots of these two types of person parameter estimates. The conventional HM simply captures the average time individuals spend on an item, potentially overlooking how individuals allocate time across test items and introducing local dependencies into the response data (Bolsinova & Tijmstra, 2018). Figure 12a illustrates that, on average, a higher speed parameter estimate was associated with a lower ability level, and vice versa (*r* = − 0.27). However, this negative relationship between the two measures may be confounded by local dependencies caused by time allocation or specific speed–accuracy strategies employed by individuals.

In contrast, Fig. 12b demonstrates that when accounting for the varying speed‒accuracy strategies of individuals, the Mix-HM-ICD adjusted the correlation coefficient to 0.14, indicating a slightly positive relationship between speed and ability. Note that, as shown in Fig. 8b, individuals in the slower-response-favoring class were able to derive greater benefits during the response process and were likely to achieve higher competency calibrations. Finally, we revisited the examination of the PPMC proportions across items to assess the fit of the Mix-HM-ICD, as shown in Fig. 7b. While not perfect, the PPMC proportions appeared to converge toward the middle value band, suggesting that the overall fit was improved by addressing potential local dependencies between responses and RTs.

**Fig. 13:**
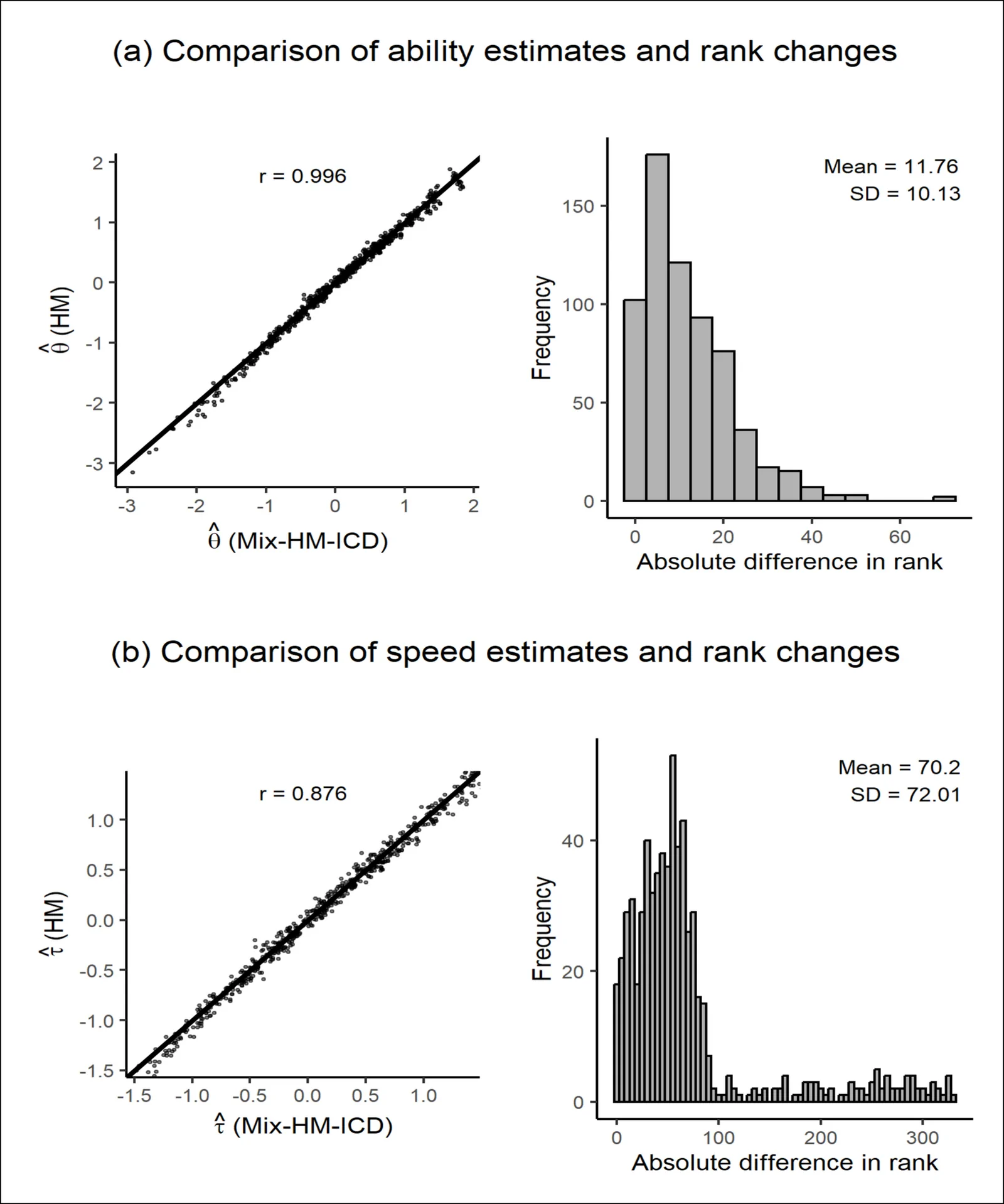
Comparison of ability and speed parameter estimates between the Mix-HM-ICD and the HM with scatter plots and rank change distributions. *Note.* HM = hierarchical model; Mix-HM-ICD = mixture hierarchical model with an invariance constraint on discrimination

To further examine differences in person-level parameter estimates between the Mix-HM-ICD and the HM, Fig. 13 presents the relationships between their estimates, along with the distributions of their rank changes. Rank changes were computed as the absolute differences in individuals’ rank positions between the two models, with higher values indicating greater ranking discrepancies.

For the ability parameter, as shown in Fig. 13a, the estimates derived from the two models were highly correlated, which was consistent with the findings obtained from the simulation study. This suggests that ignoring within-person speed heterogeneity generally leads to only modest differences in the magnitude of ability estimates. However, rank-order comparisons revealed that the HM yielded noticeable rank changes for a nontrivial proportion of the respondents when compared with the best-fitting model (Mix-HM-ICD). Although these differences may appear limited in magnitude, they can have meaningful implications in high-stakes testing contexts, where even moderate ranking shifts may affect the degree of fairness.

Turning to the speed parameter, the discrepancies were more pronounced, as illustrated in Fig. 13b. The correlation between the two models remained high but was clearly lower than that observed for the ability estimates, and the distributions of the rank changes showed substantially greater variability. These findings provide empirical evidence that ignoring within-person speed heterogeneity has a stronger impact on the speed estimation process, further highlighting the importance of accounting for individuals’ differential speed adjustments across items in joint modeling of accuracy and RT.

## Conclusion

While the HM is capable of jointly modeling responses and RTs and has gained popularity in applied analysis, there is much evidence suggesting that the relationship between responses and RTs may not be fully explained when considering the higher-level correlation between ability and speed (e.g., Bolsinova et al., 2017a, 2017b, 2017c; Molenaar & De Boeck, 2018; Mutak et al., 2024). Because individuals tend to adjust their response pace to maintain a proper balance of accuracy and speed, local dependencies between responses and RTs under the HM may interfere with the inferences of person parameters and the calibration of model parameters, making a local dependency model justifiable and imperative (De Boeck & Jeon, 2019).

This study extends the HM to allow test-takers to answer each item at their preferred pace and presents a new Mix-HM. We posit that two latent classes, faster- and slower-response favoring classes, have distinct impacts on the correctness of each item. These impacts can be thoroughly investigated through either positive or negative local dependencies. The newly developed Mix-HM is flexible and can be customized for diverse research purposes by imposing certain constraints on the model parameters. This model enables an opportunity to delve into the cognitive processes of test-taking by inspecting different types of local dependencies.

The results of Simulation 1 demonstrate that the Mix-HM achieves satisfactory parameter recovery, whereas fitting the conventional HM to data generated from the Mix-HM leads to deteriorated estimation due to neglect of local dependency between response accuracy and RT. This pattern is consistently observed across balanced, negative-dominant, and positive-dominant dependency conditions, indicating that the proposed model performs robustly under different compositions of item dependency structures at both the item and person parameter levels.

More specifically, a larger sample size improves the estimation of the model’s structural parameters, whereas a longer test enhances the precision of person parameter estimates. Although differences in ability parameter recovery between the two models are relatively small, the Mix-HM consistently shows a slight advantage. In contrast, this advantage is more pronounced for the recovery of speed parameters. Taken together, these findings suggest that the dynamic relationship between accuracy and RT cannot be adequately captured solely by the higher-level correlation between ability and speed in the HM.

The results of Simulation 2 indicate that WAIC and PSIS-LOO-CV provide effective model selection performance in terms of both hit rates and false positive rates across a range of data-generating conditions. Specifically, when the true model was Mix-HM and its variants, the CV-HM, or the HM, both criteria were generally able to correctly identify the best-fitting model within each condition.

However, when the true model was the Mix-HM, the Mix-HM-ICD exhibited a relatively higher false positive rate. To further examine, this issue, additional simulations were conducted by increasing the magnitude of the discrimination difference between the two latent classes. Under these conditions, the correct selection rates observed for the Mix-HM increased substantially, exceeding 0.90 and approaching 1.00 when the difference became sufficiently large. These results suggest that the effectiveness of model selection in distinguishing the Mix-HM from its constrained variants depends on the magnitude of parameter differences across latent classes.

Another noteworthy finding is observed when the true model is the HM, in which case the Mix-HM and its variants are frequently selected as the best-fitting models. This pattern is consistent with findings in the mixture modeling literature, where information-based criteria may favor more complex models when the penalty for model complexity is insufficient (Li et al., 2009). To address, this issue, we recommend complementing model selection criteria with parameter-level diagnostics. In particular, the latent dependency indicator ${q}_{j}$ can be examined. When the posterior probabilities of ${q}_{j}$ are close to 0.5 across MCMC samples, this indicates the absence of systematic dependency patterns for the corresponding items, suggesting that the use of the simpler HM may be sufficient despite the apparent preference for more complex models based on information criteria.

The PIAAC numeracy test was used to demonstrate the application of the proposed Mix-HM in an empirical data analysis. The PPMC evaluation indicated that most items showed poor fit under the conventional HM, suggesting potential violations of conditional independence between accuracy and RT and the presence of heterogeneous response strategies. To further examine these patterns, competing models—including the Mix-HM and its variants, the HM and its constrained version, the CV-HM, and the Mix-HM-FD—were compared. The CV-HM characterizes item dependency as a continuous function of standardized log-response time residuals, whereas the Mix-HM-FD relaxes the assumption of directional discrimination differences across latent speed classes, allowing more flexible dependency structures. Through these comparisons, the necessity of incorporating latent speed classes and flexible discrimination structures could be evaluated in an empirical context.

Based on WAIC and PSIS-LOO-CV, the Mix-HM-ICD was identified as the best-fitting model. The results indicated that most test-takers favored slower responses and that all items exhibited positive dependencies, yielding a moderate negative association between the proportion of faster-favored responses and ability. Comparisons of item parameter estimates further showed that the Mix-HM-ICD produced more stable and interpretable difficulty and discrimination estimates, whereas the CV-HM was more sensitive to extreme values and less parsimonious. In addition, the estimated relationship between ability and speed differed between the Mix-HM-ICD and the HM. Given that RT has been shown to provide valuable collateral information for improving measurement (Huang, 2020a; van der Linden, 2009; van der Linden et al., 2010), these results suggest that failing to account for latent speed heterogeneity may lead to a distorted representation of the ability–speed relationship, thereby limiting the effective use of RT information in practice.

Although the adequacy of the proposed Mix-HM can be evaluated using information-based criteria and PPMC, which assess the overall level of model fit and potential conditional dependencies, some items may not exhibit meaningful local dependence between response accuracy and RT. As noted by anonymous reviewers, forcing every item to be classified as either slow-better or fast-better may therefore be unnecessarily restrictive in certain situations.

Within the proposed framework, the Mix-HM characterizes item-level dependency through the estimation of the difference parameters ${\updelta}_{j}^{\upbeta }$ and ${\updelta}_{j}^{\upalpha }$, which reflect the direction and magnitude of dependency. Notably, this formulation does not imply that differences in item parameters between the faster and slower classes are fully explained by local dependency; rather, these parameters provide a parsimonious representation of systematic differences associated with dependency effects while allowing for the possibility that such differences may be negligible for certain items.

Consistent with the logic commonly adopted in differential item functioning (DIF) analysis, the practical importance of these effects may be evaluated using both statistical significance and substantive magnitude (Huang, 2014). Statistical significance may be assessed using Wald tests in frequentist settings or by examining whether Bayesian credible intervals exclude the null value. Beyond statistical significance, interpretation can be guided by the Educational Testing Service (ETS) DIF classification framework (Dorans & Holland, 1993; Zieky, 1993), which can be expressed in terms of odds ratios (OR), with values around 1.54 and 1.89 commonly marking the boundaries for moderate and large effects, respectively.

For item difficulty differences, the translation from OR to ${\updelta}_{j}^{\upbeta }$ is relatively direct under a logistic model, yielding approximate thresholds of ${\updelta}_{j}^{\upbeta }\approx 0.43$ and 0.64. In contrast, the interpretation of item discrimination differences is inherently more complex, as their effects depend on the ability–difficulty difference (θ − β). Under a representative condition (e.g., a baseline discrimination parameter set to α = 1 and a unit ability–difficulty difference, θ − β = 1), the same OR thresholds correspond to ${\updelta}_{j}^{\upalpha }$ values of approximately 1.43 and 1.64. These mappings provide a concise and practically interpretable reference point for evaluating the magnitude of dependency effects; however, their interpretation should be considered in conjunction with the underlying ability–difficulty relationship and the joint influence of model parameters.

It should be noted that the above thresholds are intended as heuristic guidelines rather than strict decision rules. In practice, the proposed model allows for simultaneous variation in both ${\updelta}_{j}^{\upbeta }$ and ${\updelta}_{j}^{\upalpha }$, and their joint effects on response behavior may not be fully captured by fixed cutoff values. Accordingly, researchers are encouraged to examine the implied conditional odds ratios across the latent trait continuum (i.e., as a function of θ) based on the estimated parameters. Such an approach enables a more nuanced evaluation of the extent to which invariance assumptions are violated at the item level, thereby supporting context-sensitive and substantively informed interpretations in applied settings.

A related consideration is whether local dependencies between response accuracy and RT are better modeled using mixture-based or continuous-variable approaches. In this study, these perspectives are represented by the Mix-HM and the CV-HM, respectively. The CV-HM characterizes item dependency as a continuous function of standardized log-response time residuals, providing a fine-grained representation of individual variations. In contrast, the Mix-HM models dependency through latent speed classes, offering a more structured and interpretable representation of qualitatively different response processes. Importantly, the proposed Mix-HM integrates features of both approaches: it distinguishes between speed-favoring response classes while incorporating a continuous latent parameter, namely, ${\upxi}_{i}$, to capture person-specific adaptability and within-person heterogeneity in the tendency toward faster-favored responses.

From a practical perspective, these approaches offer complementary advantages. Continuous models such as the CV-HM may be preferable when the goal is to capture subtle, continuous variation in response behavior, whereas mixture-based models such as the Mix-HM facilitate interpretation by providing discrete response categories that can support post hoc diagnostic analyses. Accordingly, the choice between these approaches should be guided by the research purpose, while the proposed Mix-HM provides a flexible alternative that balances interpretability and flexibility in modeling response processes.

In applied testing contexts, information on item-level local dependency can provide valuable diagnostic insights for improving test design. Items exhibiting positive dependency may reflect higher cognitive demands and sensitivity to time pressure, suggesting the need for adjustments in time limits or item formulation. In contrast, items showing negative dependency may indicate reliance on automated processing or lower cognitive complexity, potentially limiting their discriminative power. In addition, such dependency patterns may also reflect individual differences in response tendencies, such as cautiousness or cognitive efficiency, which should be considered when interpreting item functioning. More broadly, systematic patterns of local dependency can reveal construct-irrelevant influences, such as disengaged responses or differential time allocation strategies, and thus inform item revisions, the balancing of cognitive demands, and the design of assessments that better support valid and interpretable measurement outcomes.

The present study demonstrates satisfactory results in capturing unexpected variations caused by violations of the conditional independence assumption in the HM; however, several limitations warrant attention, and potential model extensions merit further investigation. Our modeling framework is theoretically sound and applies to a specific circumstance, but it excludes other potential influencing factors (e.g., the non-stationarity of speed and ability; see Mutak et al., 2024). As mentioned by De Boeck and Jeon (2019, pp. 6–7), the phenomenon of residual dependencies between accuracies and RTs is universal and diverse for any type of test. More fine-grained analyses are valuable for investigating the cognitive processes of individuals, suggesting that no single approach can universally address all circumstances of local dependencies. Their reminder supports our study, as the dynamic process of speed-favored responses is considered only across items in the proposed model. Efforts are needed to delve into the intricate cognitive processes involved, and advanced model development is encouraged.

For simplicity and ease of interpretation, the proposed Mix-HM does not account for the mechanism of omitted responses and treats nonresponses as missing at random. However, examinees often exhibit different test-taking behaviors in time-limited tests, spending significantly more time on certain items they attempt while skipping others due to time constraints (Pohl et al., 2019). Additionally, omitted responses may be influenced by the level of engagement of examinees, and the RT distribution can vary depending on whether an examinee engages in item responses (Ulitzsch et al., 2020). Extending the Mix-HM to incorporate the effects of omitted responses and test-takers’ engagement behaviors could enhance its generalizability and is an interesting topic for future research.

## Data Availability

The empirical dataset was derived from the Numeracy Test of the Programme for the International Assessment of Adult Competencies (PIAAC). Publicly available data can be downloaded from the OECD PIAAC Data Repository at the following URL: https://www.oecd.org/skills/piaac/data/

## Code availability

All analysis code supporting this study is publicly available on the Open Science Framework (OSF): https://doi.org/10.17605/OSF.IO/WMB5V.
